# Signal Transduction in Ribosome Biogenesis: A Recipe to Avoid Disaster

**DOI:** 10.3390/ijms20112718

**Published:** 2019-06-03

**Authors:** Manuela Piazzi, Alberto Bavelloni, Angela Gallo, Irene Faenza, William L. Blalock

**Affiliations:** 1 Istituto di Genetica Molecolare-Luigi Luca Cavalli Sforza, UOS Bologna, Consiglio Nazionale delle Ricerche (IGM-CNR), 40136 Bologna, Italy; manuela.piazzi@gmail.com; 2 IRCCS, Istituto Ortopedico Rizzoli, 40136 Bologna, Italy; alberto.bavelloni@ior.it; 3 RNA Editing Laboratory, Dipartimento di Oncoematologia, IRCCS, Ospedale Pediatrica Bambino Gesù, 00146 Rome, Italy; angela.gallo@opbg.net; 4 Dipartimento di Scienze Biomediche e Neuromotorie, Università di Bologna, 40126 Bologna, Italy; irene.faenza2@unibo.it

**Keywords:** oncology, nucleus, TP53, PI3K-AKT-mTOR, PKR-eIF2α, MYC, RNA polymerase, RNA splicing, RNA editing, translation

## Abstract

Energetically speaking, ribosome biogenesis is by far the most costly process of the cell and, therefore, must be highly regulated in order to avoid unnecessary energy expenditure. Not only must ribosomal RNA (rRNA) synthesis, ribosomal protein (RP) transcription, translation, and nuclear import, as well as ribosome assembly, be tightly controlled, these events must be coordinated with other cellular events, such as cell division and differentiation. In addition, ribosome biogenesis must respond rapidly to environmental cues mediated by internal and cell surface receptors, or stress (oxidative stress, DNA damage, amino acid depletion, etc.). This review examines some of the well-studied pathways known to control ribosome biogenesis (PI3K-AKT-mTOR, RB-p53, MYC) and how they may interact with some of the less well studied pathways (eIF2α kinase and RNA editing/splicing) in higher eukaryotes to regulate ribosome biogenesis, assembly, and protein translation in a dynamic manner.

## 1. Introduction

Ribosome biogenesis is the process by which the 47S and 5S ribosomal RNAs (runes) are transcribed, processed, and assembled with the necessary ribosomal proteins to form the small (40S) and large (60S) ribosomal subunits. Once exported to the cytoplasm, the two subunits join, in the presence of mRNA and initiator tRNA to form the pre-initiation complex (PIC) [1]. Further processing results in a mature ribosome. Ribosome biogenesis represents the most expensive, complex, finely tuned, multi-step process that the cell must carry-out; therefore, it happens to be one of the most intricately regulated and controlled. In the case of eukaryotes, the process involves the input of all three RNA polymerases (RNA pol I, RNA pol II, and RNA pol III), 79 ribosomal proteins (33 in the 40S subunit and 46 in the 60S subunit), and well over 200 proteins (helicases, splicing factors, and chaperone proteins) and non-coding RNA (ncRNA) species (miRNAs, scaRNAs, and snoRNAs) [1]. The process initiates in the nucleoli and is followed step-by-step with sequential rounds of assembly and modification of the maturing ribonucleoprotein (RNP) complexes as they migrate from the nucleoli to the nucleoplasm and ultimately to the cytoplasm, where the final assembly and maturation steps take place. Mutations in any of the necessary proteins or alterations at practically any of the maturation steps can result in dire consequences to the organism, depending on both the penetrance of the alteration and the tissue involved. Thus, ribosome biogenesis is highly regulated with diverse checkpoints to limit the production of altered ribosomes [1,2].

Additionally, the process of ribosome biogenesis is energetically expensive for the cell; its regulation must coincide with the environmental conditions in which the cell finds itself and with other cellular processes, such as cell division and differentiation. Under low nutrient conditions, ribosome biogenesis and protein synthesis would not be energetically favorable to the cell. Similarly, initiating ribosome biogenesis and protein synthesis at the same moment as cell division rather than prior to or following cellular division would be catastrophic to the cell [3,4]. This review aims to examine the major signal transduction events controlling ribosome biogenesis and the initiation of protein synthesis in higher eukaryotes. The role of well-studied pathways in ribosome biogenesis, such as the avian myelocytomatosis viral oncogene homolog (MYC)/MYC-associated factor X (MAX), mouse/human double minute 2 homolog (M/HDM2)-p53, and the phosphotidylinositol-3 kinase (PI3K)-AKR mouse thyoma homologue (AKT)-mammalian target of rapamycin (mTOR) pathways, will be reviewed as well as the roles of the less well studied eukaryotic initiation factor (eIF)-2α kinase (namely protein kinase R (PKR)) and RNA editing/alternate splicing, and how these pathways cross-talk to regulate ribosome biogenesis. Pathologies resulting from perturbations in these pathways will also be discussed.

## 2. Ribosome Biogenesis: An Overview

Ribosome biogenesis is a highly dynamic process in which transcription of the runes, processing/modification of the runes, association of ribosomal proteins (RPs) to the pre-runes, proper folding of the pre-runes, and transport of the maturing ribosomal subunits to the cytoplasm are all combined [2,5,6]. In addition to the RPs that represent the structural component of the ribosome, over 200 other non-ribosomal proteins and 75 snoRNAs are required for ribosome biogenesis. The final product is a functional ribosome, which, in eukaryotes, consists of 40S and 60S subunits that contain 4 species of processed ribosomal RNAs (18S, 28S, 5.8S, and 5S) and 79 ribosomal proteins (RPs) [2,5,6,7]. The mature 40S subunit consists of 18S rRNA and 33 RPs, while the 60S subunit consists of 28S, 5.8S, and 5S runes and 43 RPs.

**Figure 1:**
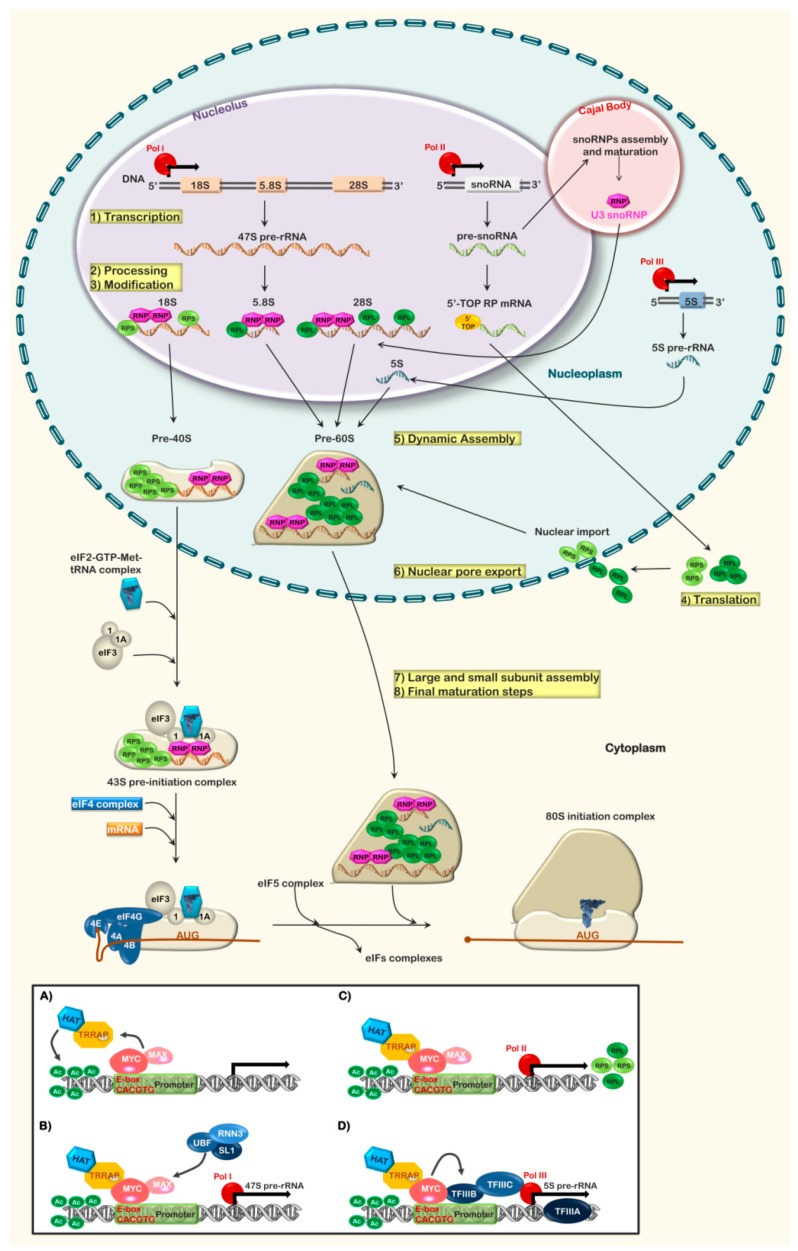
Schematic diagram of ribosome biogenesis and MYC-dependent regulation of rRNA synthesis. The diagram gives a synopsis of the steps involved in ribosome biogenesis and CAP-dependent translation with emphasis to the limiting step, rRNA synthesis.

Ribosome biogenesis initiates around nucleolar organizing regions, which contain several hundred copies of the ribosomal DNA (rDNA) genes. In humans, these genes are arranged as head-to-tail palindromes on chromosomes 13, 14, 15, 21, and 22 and encode the 47S pre-rRNA transcripts that will later be processed into 28S, 18S, and 5.8S runes [1]. In addition, on a portion of chromosome 1 (human), in association with the nucleoplasm, there are several hundred copies of the 5S rDNA gene [1]. The process begins with the association of the upstream binding factor (UBF) and selectivity factor (SL)-1 to the 47S rDNA promoter. This recruits the RNA polymerase I-specific initiation factor RNN3 (TIF-IA) and RNA pol I to the promoter. The RNA pol I complex formation is assisted by the association of the MYC:MAX heterodimer to upstream E-box elements and the binding of additional regulatory factors, which recruit the histone acetyltransferase (HAT) complex, and can be inhibited by the association of p53 or the pRB/p130 complex to key proteins of the RNA pol I initiation complex [1,8,9]. At the same time, TF-IIIA, TF-IIIB, TF-IIIC, and RNA pol III associate with the 5S rDNA promoter. The association of TF-IIIA represents the first step in the assembly of the pol III complex, by both inducing a minor bend in the DNA as well as assisting in the incorporation of TF-IIIC into the polymerase complex. TF-IIIB, in turn, induces a major bend in the DNA at the transcriptional start site [10,11]. Again, this complex formation is assisted by the association of MYC with TF-IIIB, in the absence of MAX, and the recruitment of the HAT complex, and either p53 or the pRB/p130 complex can suppress RNA pol III-mediated transcription (Figure 1) [8,9]. The 18S, 5.8S, and 28S runes are transcribed by RNA pol I as a single precursor RNA from tandem repeats of the gene into the nucleolus, while the 5S rRNA, which is transcribed by RNA pol III from multiple genes into the nucleoplasm, migrates to the nucleolus. RNA pol III is also responsible for the transcription of tRNA genes needed later for translation initiation and elongation. In contrast, the ribosomal proteins, which are present throughout the genome (present on 20/23 chromosomes counting the sex chromosomes) are transcribed by RNA pol II in association with MYC:MAX and the recruitment of the HAT complex to the promoter [1,8,9]. The mRNAs encoding the RPs are processed and transported to the cytoplasm for translation. RNA splicing of these transcripts also produces the scaRNAs and snoRNAs later needed for the formation of diverse heterogeneous ribonucleoprotein (hnRNP) complexes, C/D snoRNPs, and H/ACA snoRNPs that function in mRNA splicing and rRNA modification/maturation (Figure 1) [2,12]. Alternative splicing of RP transcripts also produces “pseudogenes” that regulate the expression/accumulation of RPs (see section on RNA editing and splicing) [13]. Newly translated RPs are then actively imported from the cytoplasm to the nucleolus and nucleoplasm where they are incorporated into the assembling ribosome. This process requires the presence of diverse chaperone proteins that serve several functions: (1) Protect the RPs from degradation, (2) facilitate their active nuclear import, and (3) assume the correct incorporation of the RPs into the maturing ribosome subunits. Recent developments in cryogenic-electron microscopy (cryo-EM) have shed a tremendous amount of light on the process of RP nuclear import and their incorporation into the ribosome. For further information on this aspect of ribosome biogenesis, the reader is encouraged to see the following publications: [12,14,15,16,17,18,19] (Figure 1).

The initial 47S pre-rRNA transcript maintains a secondary structure at the newly synthesized 5′-end that acts as a platform for the binding and association of an initial set of RPs forming the 90S RNP. As the 5’ portion of the pre-rRNA contains what will become 18S rRNA of the 40S subunit, the RPs associating with the 5′-end are small subunit RPs or RPSs. Two of the first RPs to associate with the pre-rRNA at the 5′-end are RPS7 and RPS24, which are required to initiate processing and cleavage of the pre-rRNA at the 5′-external transcribed spacer (ETS) (Figure 1). The initial binding of the RPSs and the processing at the 5’-ETS, as well as modifications of the rRNA by ribose methylases (C/D box snoRNPs) and polyuridylases (H/ACA box snoRNPs), energetically favors an rRNA structure that forms a platform for the next round of RPs to associate [2,5,6]. At the same time that processing of the 5′-end of the 18S rRNA is being conducted, association of RPS17 and RPS19 facilitate the processing and cleavage at the 3′-end of the 18S rRNA within the internal transcribed sequence (ITS1), liberating the assembling 40S subunit from the 60S subunit [2,5,6]. Low levels of RPS7 or RPS24 block or retard rRNA processing, resulting in failed maturation of the 5′-end of the 18S rRNA. Similarly, depletion of RPS17 or RPS19 result in failed processing of the 3’-end of the 18S rRNA. Virtually the same process occurs for the formation of the maturing 5.8S and 28S with the ITS2 (between 5.8S and 28S sequences). At this point, the associating RPs are RPLs as they will become part of the 60S subunit. The process, marked by rounds of rRNA processing and folding and rounds of RP association, continues to occur until the last of the RPs for each subunit are finally incorporated in the cytoplasm [2]. Due to the ability of RNA to assume diverse energy structures at equilibrium, diverse modes of RNP assembly can be occurring simultaneously, including the synthesis of kinetically dead-end products that are aborted and targeted for degradation and recycling by the TRAMP4/5 complex [2,6]. The enzymes and proteins that modify and cleave the 47S rRNA are numerous and the process complex; we invite the reader, for more detail in this aspect of rRNA processing, to refer to the following reviews: Turowski and Tollervey, 2015; Fernandez-Pevida et al., 2015; and Mullineux and Lafontaine, 2012 [20,21,22].

Several lines of evidence indicate that the main purpose of the RPs is to stabilize rRNA folding and structure, thus assisting in processing; (i) the RPs are RNA-binding proteins that are dependent on RNA structure and conformation, (ii) there are very few protein–protein interactions between the RPs, (iii) while many of the RPs possess a tail that inserts into the ribosome core, the core is, proteically speaking, mostly hollow, with the exception of the rRNA species; and the RP tails apparently do not interact with the mRNA substrate that will later occupy the core [2,5]. In addition to the RPs incorporated into the ribosome and the rRNA modifying proteins, a number of proteins associate with the maturing ribosome in the nucleus to ensure that the ribosome does not prematurely assemble, including the Shwachman–Diamond Syndrome associated protein, SBDS, and eukaryotic initiation factor 6 (eIF6). Once exported into the cytoplasm, these chaperons disassociate from the ribosomal subunits, allowing for ribosomal subunit assembly and the association of mRNA and translation initiation factors (IFs) to form the PIC [2,5,6,23,24]. Final steps involving the eIF2, eIF4, and eIF5 complexes result in mature ribosomes and the initiation of protein synthesis. Alteration at any step of this process can have mild to dire consequences depending on the defect and its penetrance. Tissues, which have the greatest rates of proliferation and turn-over, such as the skin and hematopoietic precursors, especially of the erythroid lineage, are often the most affected (Figure 1).

Many RPs also have other trans-regulatory functions [2,25]. Processing of the 60S subunit requires the incorporation of the 5S rRNA, which initially associates with RPL5 and RPL11 to form the 5S RNP. Under the conditions of RP haploinsufficiency or disturbances in ribosome biogenesis as a result of altered signaling or stress, the process of pre-rRNA maturation is arrested, resulting in an increasing pool of unassimilated RPs. The accumulation of free RPs, namely the 5S RNP, results in the inhibition of protein synthesis and cell cycle arrest through the activation of p53 [2,25,26]. Other than enhanced p53 expression, other mechanisms may explain the pro-apoptotic and poor proliferative state observed, following the loss or reduced expression of RPs. Loss of RPL5 or RPL11 was shown to lead to reduced cyclin expression, and therefore, reduced proliferation. Additionally, cells deficient in RPS19 and RPL11 were demonstrated to be predisposed to oxidative stress [2,25]. In addition, an innate immune component has been observed. RP loss/reduction enhances the expression of innate immune genes including interferon and TNFα, which is known to contribute to the hematopoietic failure in RPS19 deficiency, likely through IRES-mediated translation [2,26].

Technological advances in microscopy have made it clear that the cell goes to great lengths in order to compartmentalize the phases of ribosome biogenesis. The nucleolus contains several sub-compartments: The fibrillar centers (FCs), the dense fibrillar centers (DFCs), and the granular compartments (GCs). In addition, on the nucleolus/nucleoplasm border there are the Cajal bodies, a distinct subcellular component responsible for non-coding RNA maturation [27,28]. The rDNA clusters are located at the FC/DFC interface and thus 47S rRNA synthesis takes place here, while 5S rRNA synthesis occurs in the nucleoplasm, but is then transported to the nucleolus. Many of the early steps in pre-rRNA maturation and pre-RNP (90S and 5S RNPs) assembly occur in the DFC, with later assembly steps requiring the incorporation of additional RPs and rRNA modification by snoRNAs, occurring in the GC. The final RPs are incorporate to form the 40S and 60S subunits in the nucleoplasm, prior to the export of the individual subunits to the cytoplasm. Interestingly, the Cajal bodies have no role in direct ribosome biogenesis but provide an environment for the maturation/modification of the snRNPs (later required for splicing) and snoRNPs (later required for rRNA processing). These sub-nuclear compartments are defined by their respective protein content with the fibrillar centers (FC/DFC) containing the methyltransferase, fibrillarin, and the Cajal bodies containing coilin. It has been proposed that the exchange between these compartments is free (no active transport necessary), as no membrane exists to define or separate them [27,28]. In this “free exchange model” the composition and ratio of water-soluble nucleic acids to insoluble nucleic acid binding proteins produces a semi-solid plasma state allowing for the free movement of protein–nucleic acid complexes between these compartments. Thus, it would be predicted that much of the order of these compartments is dictated by the localized protein content.

## 3. MYC a Global Regulator of Ribosome Biogenesis

It is estimated that approximately 15% of genes in higher eukaryotes contain MYC-responsive regulatory elements; thus, it is no wonder that overexpression of MYC can induce uncontrolled protein synthesis and cell proliferation. MYC is one of the only transcription factors known to regulate all three of the RNA polymerases (pol I, pol II, and pol III) and, therefore, has the capacity to induce the expression of all required rRNAs, ribosomal proteins, and co-factors necessary for ribosome biogenesis [8,29]. In complex with the MAX protein, MYC binds to E-box elements upstream of the transcriptional start site in the promoter of MYC-responsive genes. Binding of the MYC:MAX heterodimer results in the recruitment of co-regulatory proteins, such as the transformation/transcription domain-associated protein (TRRAP) to the promoter. TRRAP is part of the histone acetyltransferase (HAT) complex, which is responsible for targeting acetylation of histones (H3 and H4) through the activity of the GCN5 acetyltransferase, thereby opening the DNA for transcription (Figure 1, bottom panel a,c). In the case of rDNA, MYC also results in the recruitment of RNA pol I co-factors UBF and SL-1 to the promoter, thus stimulating the transcription of the 47S pre-rRNA (Figure 1, bottom panel b) [8,30,31]. In contrast, MYC influences the transcription of the 5S rRNA and that of tRNAs in a diverse manner. Rather than forming a heterodimer with MAX, MYC associates directly with TF-IIIB in the nucleoplasm to stimulate RNA pol III-mediated transcription of theses RNAs (Figure 1, bottom panel d) [8,32]. Beyond the direct association with rDNA promoters, MYC is also known to influence the expression of the RNA pol I transcriptional co-factor UBP, a MYC-responsive gene product [8,33].

MYC is also known to induce the expression of both the small and large subunit ribosomal proteins, in an RNA pol II-dependent manner. Interestingly, several of these targets RPL5 and RPL11 have been found to be extremely important in sensing ribosomal stress (see below). RPL11 has been shown not only to induce p53 transactivation, but also to bind MYC within the MYC box II domain and inhibit its association with TRRAP, thereby reducing histone acetylation and MYC-dependent transcription [8,34]. Thus, the MYC-RPL11 circuit functions in a negative-feedback mode. In addition, MYC also induces a number of proteins that are either involved in rRNA processing and transport or in translation initiation. MYC controls the expression of nucleolin (NCL) and nucleophosmin (NPM), two proteins that are involved in multiple processes in the nucleus, including the processing of the 47S rRNA to 18S, 5.8S, and 28S rRNAs; as well as the expression of the nucleolar protein 56 (NOP56), a core component of the C/D box snoRNP complex, block of proliferation 1 (BOP1), part of the PeBoW complex required for 28S and 5.S rRNA maturation, and dyskerin (DKC), a H/ACA snoRNP complex subunit responsible for the pseudouridylation of rRNA species [8,35,36]. Moreover, NPM has additional roles in the cytoplasmic-nuclear import of newly synthesized ribosomal proteins and the nuclear-cytoplasmic export of the assembling ribosomal subunits. MYC also enhances expression of the translation initiation factors eIF2α, eIF4A-I, eIF4E, and eIF4G, which regulate CAP-dependent translation and may also promote methylation of the mRNA CAP through RNA guaine-7-methytransferase [8,37].

It is now well established that MYC transformation is heavily dependent on an altered rate of protein synthesis. Studies document enhanced protein synthesis in Eμ-MYC transgenic mice, which rapidly develop tumors. When these mice were crossed with mice containing haplodeficiencies of the genes encoding RPL24 or RPL38, the rate of lymphogenesis was substantially reduced, indicating that reduced protein synthesis, due to the reduce expression of a necessary RP, was able to contrast MYC transformation [38].

Under certain circumstances, MYC is also known to promote apoptosis. Diverse forms of MYC can be expressed by alternate translation initiation. Two main forms, p64 and p67, result from alternate start codon usage. MYC p64 initiates from a standard AUG start codon; in contrast, MYC p67 initiates from a non-canonical upstream start codon (a CUG), which produces a protein that is 15 amino acids longer. Both these forms can associate with E-box elements, while MYC p67 can also associate with CAAT-enhancer binding elements as well, thus affecting the transcription of an additional set of genes [39,40]. It has been proposed that the ration of p64 MYC to p67 MYC dictates whether MYC promotes growth/proliferation or apoptosis.

## 4. The PI3K-AKT-mTOR Pathway, Linking Ribosome Biogenesis to Extracellular Signaling

**Figure 2:**
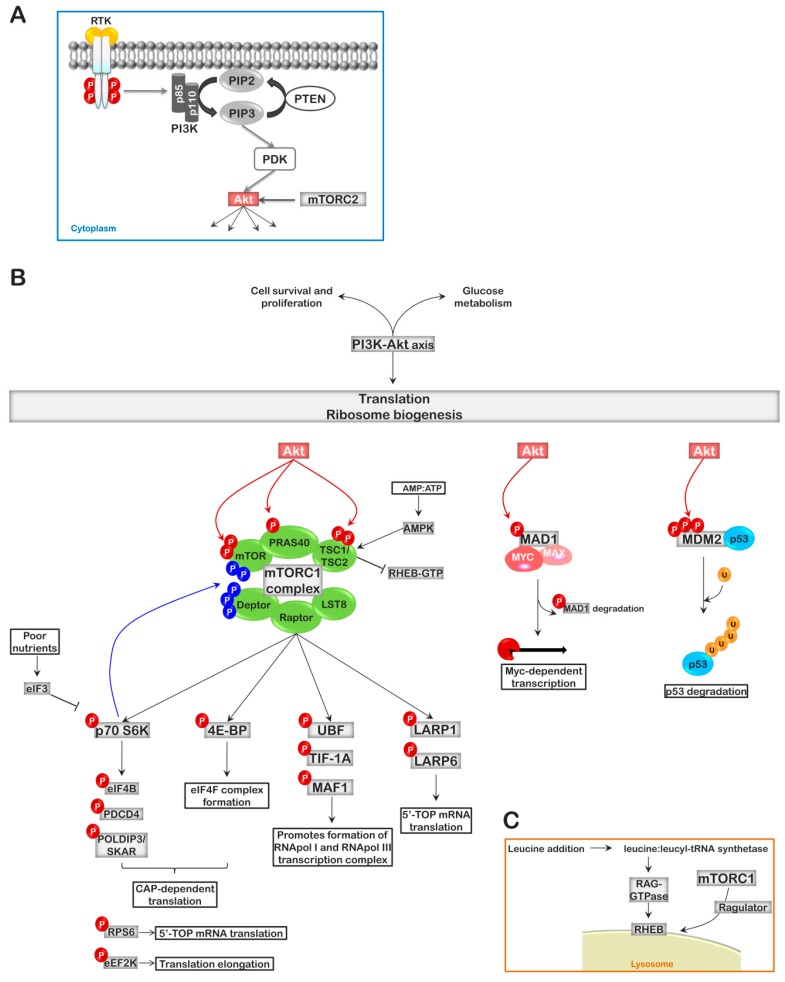
Diagram of the regulation and role of the PI3K-AKT-mTORC1 pathway in ribosome biogenesis and translation initiation. The diagram presents the diverse points of regulation that the PI3K-AKT-mTORC1 signaling pathway has in ribosome biogenesis and translation initiation under optimal as well as suboptimal (low ATP levels, poor nutrients, limited amino acid) conditions. (**A**) Activation of AKT through the regulation of PIP3 levels; (**B**) AKT stimulates the mTORC1 complex, which targets multiple downstream targets; (**C**) l-leucine activation of mTORC1 at the lysosome. Phosphorylation (P) marked in red represent phosphorylations that favor ribosome biogenesis and translation initiation; phosphorylations in blue represent phosphorylations that are inhibitory to ribosome biogenesis or CAP-dependent translation initiation; ubiquitinations are presented in orange.

The PI3K/AKT pathway has become one of the most studied and best characterized signal transduction pathways, due to its involvement in cell survival and proliferation, glucose metabolism, and translation [41]. A large number of cytokine and growth factor receptors, such as the epidermal growth factor receptor (EGFR), the insulin-like growth factor receptor (IGFR), the granulocyte macrophage-colony stimulating factor receptor (GM-CSFR), and the tumor necrosis factor (TNF)-α receptors (TNFR1 and TNFR2) transduce part of their signal through the PI3K-AKT-mTOR pathway. These receptors inform the cell of the surrounding environment, whether to undergo self-renewal or differentiation. In the classical scenario, following ligand binding to its cognate receptor, the regulatory and catalytic domains of the phosphatidylinositol-3 kinase (PI3K) are recruited to the cytoplasmic domain of the receptor and activated. Activated PI3Ks catalyzes the phosphorylation of phosphatidylinositol (PtdIns), PtdIns(4)P, PtdIns(5)P, or PtdIns(4,5)P2 at the 3-position of the inositol ring to form PtdIns(3)P, PtdIns(3,4)P2, PtdIns(3,5)P2, and PtdIns(3,4,5)P3 [41,42]. PtdIns(3,4,5)P3 is the main form involved in AKT activation, and its level in cells is regulated by the phosphatase and tensin homolog (PTEN), the product of the *mmac1* gene, which quickly dephosphorylates PtdIns (3,4,5)P3 to PtdIns(4,5)P2. It is not surprising that the gene encoding PTEN is one of the genes most often mutated or lost in cancer [43]. PtdIns(3,4,5)P3 results in the recruitment of the AKT kinase (AKT1, -2, or -3) via the N-terminal and negative regulatory plekstrin homology (PH) domain. This association not only results in the localization of AKT to membrane components of the cell, but it also causes a conformational change in AKT, removing the negative regulation imposed by the PH domain, opening up AKT for two phosphorylation events required for its kinase activity. The phosphatidylinositol-dependent kinase, PDK1 is responsible for phosphorylating AKT1 on Thr308 (Thr309 on AKT2 and Thr308 on AKT3). Like AKT, it is recruited to the membrane via its PH domain. Phosphorylation on Thr308 is assisted by phosphorylation of Ser473 in AKT1 (Ser474 in AKT2 and Ser472 in AKT3), which is carried-out by the mTORC2 complex (Figure 2A) [44].

While the AKT kinases show some redundancy in their activity, several major differences have been noticed. AKT1 and AKT2 are ubiquitously expressed, and both are present and have enzymatic roles in the nucleus and cytoplasm. In contrast, the localization of AKT3 is predominantly nuclear, with expression limited to the brain, lung, and kidney in adults; and heart, liver, and brain in fetus. AKT1 is associated more closely with anti-apoptotic/survival effects of PI3K activation, while AKT2 has been shown to be responsible for AKT-dependent insulin signaling. AKT3 is still poorly understood with few substrates identified [45,46].

Multiple substrates of AKT have been identified, but the best understood and most critical by far to ribosome biogenesis is the mTORC1 complex. The mTORC1 and mTORC2 complexes differ in several ways. First, mTORC1 serves as a substrate for AKT, while mTORC2 is responsible for the phosphorylation of AKT on Ser473. Second, mTORC1 is a rapamycin-sensitive complex while mTORC2 is not. This difference in susceptibility to rapamycin is due to the third major difference in these complexes; the protein components. Both mTORC1 and mTORC2 contain the mammalian target of rapamycin protein (mTOR), the positive/negative regulator G protein β-subunit-like (GβL or LST8), and the DEP-domain containing mTOR-interacting protein (Deptor), a negative regulator of the mTORC complexes; but while mTORC1 contains the scaffolding protein Raptor, mTORC2 contains Rictor, Sin1 (MAPKAP1), and proline-rich protein 5 (PRR5). The mTORC2 complex is nutrient insensitive, acts upstream of Rho-GTPases, and has a role in modifying the actin cytoskeleton. In contrast, the mTORC1 complex is nutrient sensitive and regulates a major part of ribosome biogenesis and CAP-dependent translation (Figure 2B) [42].

The AKT kinases can directly phosphorylate mTOR (Thr2446, Ser2448), which increases the activity of the catalytic subunit, mTOR, but is not sufficient for mTORC1 activation. Activation of the mTORC1 complex occurs through its GTPase Rheb when in the GTP bound state. Rheb is inhibited by its GAP protein(s), the tuberous sclerosis heterodimer (TSC1/TSC2). In addition, the mTORC1 complex is also inhibited by the association of the proline-rich AKT substrate (PRAS40) in a 14-3-3 protein-dependent manner. AKT activates the mTORC1 complex by phosphorylating both PRAS40 (Thr246) and the TSC1/TSC2 (Ser939, Ser981, and Thr1462 of TSC2) complex to free Rheb and stimulate the activation of mTORC1 (Figure 2B) [42,47].

Amino acid activation of mTORC1 is also possible. This involves the recruitment of mTORC1 by the Ragulator protein complex to the lysosomal membrane, following stimulation with amino acids, where mTORC1 interacts with its activator Rheb, bringing the mTORC1 complex in contact with the Rag GTPases. The heterodimeric Rag GTPases, consisting of RagA or RagB pairing with RagC or RagD, become loaded with GTP in the presence of amino acids, favoring their interaction with Raptor and the activation of mTORC1 [48,49,50]. Nicklin et al. demonstrated that it was glutamine uptake and its subsequent efflux in the presence of essential amino acids, which is the limiting step in this process [48,51]. The uptake of glutamine by the cell establishes an internal reservoir of glutamine that can be exported by the heterodimeric SLC7A5-SLC3A2 antiporter. The efflux of glutamine by the antiporter promotes the import of branched-chain amino acids such as leucine [48,51]. This increased presence of intracellular leucine favors the interaction of leucine with leucyl-tRNA synthetase. The leucine:leucyl-tRNA synthetase complex then acts as a GTPase-activating protein stimulating the Rag GTPases [48,52]. So why leucine? Leucine happens to be the amino acid most frequently used in proteins, thus, its deficiency should set-off alarms for the cell. For this reason, it has been observed that some ribosomopathy patients can be treated with leucine supplements (see below). Moreover, the presence of Raptor in the complex assists in the recruitment of mTORC1 substrates (Figure 2C).

Finally, the activity of the mTORC1 complex can be regulated directly by the energy level of the cell. Low cellular ATP levels result in the activation of the adenosine monophosphate activated kinase (AMPK), which can phosphorylate and activate TSC2, of the TSC1–TSC2 inhibitory complex, on Thr1227 and S1345; and/or phosphorylate Raptor on Ser722 and Ser792, promoting its interaction with 14-3-3 proteins and the inhibition of mTORC1 (Figure 2) [53,54]. In addition, AKT has the ability to autoregulate its phosphorylation at Ser473 through the phosphorylation Sin1 (Thr86) to down-regulate mTORC2 activity [55].

The mTORC1 complex phosphorylates two main targets of ribosome biogenesis, the S6 kinases (p70 S6K1/p70 S6K2) and the eIF4E-binding protein (4E-BP). Recruitment of the 40S ribosomal subunit to the 5′ N^7^-methyl guanosine CAP [m^7^G(5’)ppp(5’)N] of mRNA is facilitated by the eIF4F translation initiation complex, which is composed of the cap-binding protein eIF4E, the scaffold protein eIF4G, and the RNA helicase eIF4A. Unphosphorylated 4E-BP associates with eIF4E, blocking the association of eIF4E with eIF4G. Phosphorylation of 4E-BP, mediated by mTORC1, frees eIF4E, facilitating its association with eIF4G and the formation of the eIF4F complex; thus, favoring CAP-dependent translation. The reduced efficiency of the eIF4F complex to recognize and promote the translation of 5′ N^7^-methyl guanosine CAPed mRNAs favors internal ribosome entry site (IRES)-mediated translation, which is often observed during inflammation and stress [42,56]. On the other hand, phosphorylation of the p70 S6 kinase, results in its activation and the subsequent downstream phosphorylation of PDCD4 (Ser67; an inhibitor of eIF4A), causing its ubiquitination and proteolysis, and eIF4B (Ser422; an activator of the eIF4A helicase), thus, favoring CAP-dependent translation of mRNAs with complex secondary structure at the 5′-end [57,58,59]. Additionally, p70 S6K also phosphorylates polymerase delta-interacting protein 3 (POLDIP3/SKAR) on Ser383 and Ser385, favoring nuclear export and translation of spliced over non-spliced mRNAs [60]. Under poor nutrient conditions, the eIF3 initiation complex associates with p70S6K and sequesters it in an inactive state. Following the appropriate stimulus, eIF3 is released and p70 S6K phosphorylates its targets (Figure 2B) [61].

Beyond CAP-dependent translation initiation, the AKT-mTORC1-p70 S6 kinase pathway has been demonstrated to target other effectors of ribosome biogenesis. AKT has been shown to phosphorylate MAD1 on S145, resulting in its release from the MYC/MAX/MAD1 heterotrimer, and its degradation to form the active MYC/MAX heterodimer; thus, promoting MYC-dependent transcription [9,62]. AKT also phosphorylates and stabilizes MDM2 (S166, S186, and S188), favoring the degradation of p53, a major repressor of ribosome biogenesis (see below). In addition, AKT may regulate the type of mRNA translated based on its 5′ UTR through phosphorylation of LARP6 (S451), a protein that associates with stem-loops in the 5′UTR to stabilize mRNA. Phosphorylation of LARP6 at S451 results in LARP6 degradation [63]. Likewise, mTOR phosphorylates LARP6 (S340, S409) and its family member LARP1 (S766, S774), but in contrast to AKT-dependent phosphorylation, mTOR-dependent phosphorylation promotes the stability and sequestering of these proteins; thus, favoring the translation of mRNAs containing the 5′terminal oligopyrimidine (TOP) motif, a 5’-cytidine followed by a short pyrimidine tract (4-14 nucleotides) immediately downstream of the methyl guanidine cap (m^7^Gppp) (Figure 2B). The 5’TOP mRNAs encode components of ribosome biogenesis such as the RPS and RPL proteins [64,65]. Moreover, mTORC1 is able to stimulate RNA pol I-dependent transcription of the 47S rRNA by activation of UBF and TIF-1A; and RNA pol III-dependent transcription of 5S rRNA and tRNA through its direct recruitment to the promoter, by TF-IIIB, and subsequent phosphorylation of MAF1 (S60, S68, and S75), an inhibitor of the TF-IIIB complex formation; thus, establishing a role for mTORC1 as a transcription factor. Use of the mTOR inhibitor rapamycin blocks the synthesis of rDNA by inhibiting the formation of the RNA pol I and RNA pol III transcription complexes on their respective promoters [9,66,67]. It is also apparent that mTOR may also regulate the balance between mTORC1 and mTORC2 complex formation by phosphorylating diverse components of the mTORC complexes, including itself. Finally, the p70 S6 kinases also phosphorylate RPS6 and the eukaryotic elongation factor 2α (eEF2α) kinase (eEF2K) [68,69]. Phosphorylation of RPS6 results in the enhanced translation of 5′TOP RNAs, while phosphorylation of eEF2K results in inhibition of its catalytic activity and the activation of eEF2α, favoring translation elongation. RPS6 can also be phosphorylated by p90^RSK^, which is activated downstream of the RAS-RAF-ERK pathway activation [70]. In addition to these, p70 S6K appears to also regulate AKT and mTORC1 activity through phosphorylation of Thr2446 and Ser2448 of mTOR and the phosphorylation and targeted degradation of Deptor (Ser286, Ser287, and Ser291) and Rictor (Thr1135). The phosphorylation of Rictor is considered a negative feedback modification as it results in decreased mTORC2 phosphorylation of AKT [71,72,73].

Recently, Bavelloni et al. reported a study in which they sought to identify novel nuclear AKT substrates. Using phospho-AKT substrate specific antibodies coupled with mass spectrometery analysis, the authors identified a set of proteins present in the nuclear lysates of two hematopoietic cell lines that were immunoprecipitated with antibodies recognizing the following epitopes: K/R-x-K/R-x-x-S^*^/T^*^ or R-x-x-S^*^/T^*^; where “x” represents any amino acid and the asterisk represents a phosphorylated amino acid. The authors then analyzed the identified proteins to determine if they actually contained sites that could be recognized by the antibodies employed in the study. Both AKT and p70 S6K belong to the AGC kinase family and have similar phosphorylation consensus sites; thus, the identified proteins may represent both AKT and p70 S6K substrates (Table 1). Many of the identified proteins are intimately related to ribosome biogenesis [74]. Thus, it is possible AKT-mTOR-p70 S6K signaling has additional targets that influence ribosome biogenesis and translation initiation that have yet to be characterized.

## 5. Cell Cycle Regulators and Ribosomal Stress

**Figure 3:**
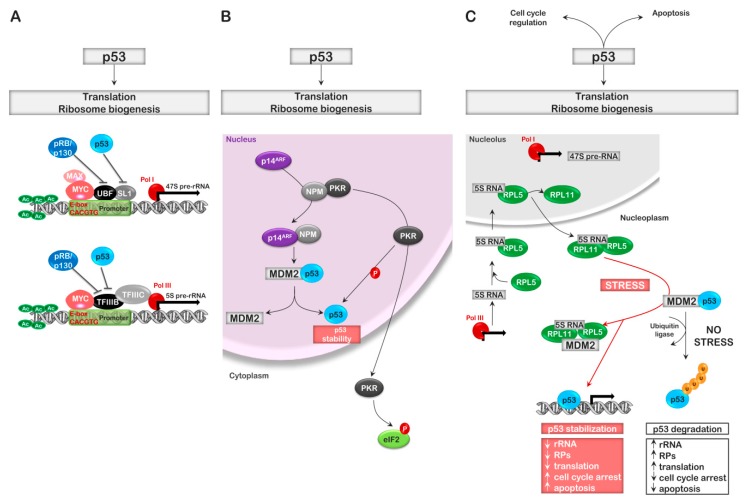
Regulation of ribosome biogenesis by cell cycle regulators. (**A**) The cell cycle regulators/tumor suppressors pRb/p130 family and p53 regulate ribosome biogenesis by suppressing rDNA transcription initiation. (**B**) The tumor suppressor p14^ARF^, an alternate open reading frame of the p16^INK4A^ gene, regulates ribosome biogenesis and translation initiation through its interaction with NPM1 and MDM2, resulting in p53 stability and transactivation and eIF2α phosphorylation. (**C**) Faulty assembly of ribosomal proteins (RPS and RPL) result in elevated levels of the pre-assembly RNP complex 5S RNP (RPL5-RPL11-5S rRNA), which binds to MDM2 resulting in p53 stability and transactivation. Additional RPSs and RPLs have been reported to bind MDM2 as well, resulting in the same effects on p53.

Coordination between cell division and proteins synthesis is imperative for cell survival; thus, it is not surprising that multiple regulators of the cell cycle also have a significant role in controlling ribosome biogenesis. Unphosphorylated retinoblastoma protein (Rb) family members not only regulate the cell cycle by associating with the E2F transcription factor, but their hypophosphorylated forms also directly associate with UBF of the RNA pol I complex and TF-IIIB of the RNA pol III complex, inhibiting the synthesis of the 47S and 5S rRNAs, as well as the necessary snoRNAs and tRNAs (Figure 3A). Loss of Rb expression or hyperphosphorylation of Rb, due to the activation of the cyclin-dependent kinase (CDK)-cyclin complex, results in the removal of this level of control [9,75,76,77]. Additionally, the smaller of the products of the *ink4a* tumor suppressor gene, p14^ARF^ (p19^ARF^ in mice) also associates with proteins of the RNA pol I complex affecting both 47S rRNA transcriptional initiation and termination [78]. More interestingly, p14^ARF^ has a significant role in regulating both rRNA processing, as well as p53-dependent transcription through its association with nucleophosmin (NPM1). NPM1 is a highly expressed nuclear phosphoprotein involved in diverse cellular processes (rRNA processing, ribosome protein nuclear import, ribosome assembly, and ribosome subunit nuclear export). NPM1 associates with diverse proteins, influencing their activity; among these are the p53 ubiquitinase MDM2 and the dsRNA-dependent inflammatory/stress activated kinase, PKR. When bound to NPM, these proteins are sequestered to the nucleus. Enhanced expression of p14^ARF^ results in its association with NPM and the formation of an MDM2 inhibitory complex, thus stabilizing p53 [79,80,81,82,83]. Similarly, the sequestration of PKR, by NPM, keeps it localized to the nucleus where its localization is associated with cell growth and DNA repair (Figure 3B). Garcia et al. reported that, following viral infection, enhanced expression of p14^ARF^ promoted its association with NPM, resulting in the release of PKR and the translocation of PKR to the cytoplasm, where it phosphorylates eIF2α, resulting in the inhibition of protein synthesis (see below) [84].

The p53 transcription factor is a master regulator of the cell. Most of the genes induced by p53 are involved in cell cycle regulation (arrest) and apoptosis; therefore, stimulation of p53 leads to cell cycle arrest and repair or subsequent cell death in most cases. In addition, p53 shares the stage with two closely related family members, p63 and p73. These family members may cooperate with or antagonize one another, depending on the promoter and the gene in question [85]. Approximately 50% of human tumors contain mutant p53. These mutations are known to affect the transactivation capacity of p53, p53 stability, and the ability of p53 to interact with additional cofactors [9,85].

The status of p53 is also extremely important in the regulation of ribosome biogenesis. The association of p53 with SL-1 complex of RNA pol I or TF-IIIB of the RNA pol III complex results in transcriptional repression of these rRNA, tRNA, and snoRNA genes. The interaction of p53 with RNA pol II-dependent promoters can either stimulate or repress their transcription [9,86,87]. As stated above, p53 protein levels are chiefly regulated at the level of protein stability. The E3 ubiquitin ligase MDM2 associates with p53 and ubiquitinates it, thereby targeting it for degradation by the proteosome. Interestingly, p53 binding of the *mdm2* gene stimulates the synthesis of its transcript, thus p53 can autoregulate its expression through the induction of MDM2. The mechanisms that regulate ribosome biogenesis have taken advantage of the MDM2-p53 relationship as a checkpoint for ribosomal stress. Alterations in the levels of proteins required for rRNA synthesis, processing, and transport can influence the MDM2-p53 interaction [79,88]. Thus, alterations that may impinge on the early steps of ribosomal biogenesis also influence p53 stability, favoring the accumulation of p53. Among these proteins are NPM1 (see above) and nucleostemin (NS). Overexpression of NS causes its accumulation in the nucleoplasm and its association with MDM2 via interaction of the coiled–coiled domains of NS with the acidic domain of MDM2, thus inhibiting p53 ubiquitination and enhancing p53 stability. In contrast, depletion of NS activates p53 through the ribosomal protein pathway [79,89].

Additionally, the accumulation of rRNAs must match the level of rRNA processing protein complexes, which must match the synthesis of ribosomal proteins to be incorporated into the assembling ribosome, which must match the transport/chaperone proteins available. A certain amount of leeway must be inherent in the system and controlled or “tweaked” through transient activation stimulation of key signal transduction pathways. The system must also have the ability to alter the assembly of the ribosome to favor the translation of certain mRNAs over others when necessary for the cell. The obvious disproportion of the necessary RNA or protein intermediates results in the stimulation of p53 transcriptional activity, arresting the process of ribosome biogenesis. This safety switch for the organism impedes the production of ribosomes that possess altered activity, which could be deleterious to the organism (constitutive p53 activity can also contribute to disease—see below). Several ribosomal proteins are known to bind to MDM2 and inhibit its activity toward p53; these include RPS3, RPS7, RPL5, RPL11, and RPL23 (Figure 3C) [79,88].

The ribosome proteins are produced in excess in the cytoplasm. The stability of these proteins is dependent on their interaction with chaperones and nuclear import proteins. Those RPs not associated with chaperones and directed to the nucleus for ribosome assembly are quickly degraded by the ubiquitin proteosome complex (UPC). Thus, free nucleolar/nucleoplasm accumulation of these RPs would signal a failure in the maturation process of the 40S and 60S subunits and stimulate p53. One of the more interesting complexes is the RPL5-RPL11-5S rRNA (5S RNP). This complex, which has an early and critical role in rRNA processing, is an early sentinel for defects in ribosome biogenesis. Bursac et al. and Sloan et al. demonstrated that RPL5-RPL11-5S rRNA accumulation and association with MDM2 could take place in both the nucleolus and nucleoplasm [90,91]. Interestingly, the RPL5-RPL11-5SRNP complex is also likely responsible for p53 accumulation in response to the deficiency of many of the additional ribosomal proteins that do not directly interact with MDM2 (Figure 3C).

## 6. EIF2α Regulation and Translation Initiation: The PKR Story

The ultimate goal of ribosome biogenesis is to produce ribosomes capable of accurately and successfully translating mRNAs into protein. Like ribosome biogenesis, the process of translation has a rate-limiting step, which is initiation; therefore, in addition to the eIF4F translation initiation complex, which is under the control of the AKT-mTOR and RAS-RAF-MAPK pathways, two additional initiation factors represent major points of translation control, eIF2 and eIF2B. These complexes bare both the GTP and the Met-tRNA necessary for pre-initiation complex (PIC) formation and translation initiation, as well as the proteins for the GDP to GTP exchange required to initiate the next round of translation [92,93,94]. Regulation of eIF2 is via the α-subunit (eIF2α) and is probably the best understood mechanism regulating translation initiation. One of four different kinases (PKR, PERK, GCN2, or HRI) leads to the phosphorylation of eIF2α. Phosphorylation of eIF2α on Ser51 results in eIF2 being locked in the GDP bound state with eIF2B, unable to catalyze the initiation of protein synthesis. As the eIF2 complex is limited compared to eIF2B, it does not take much phosphorylated eIF2α to soon result in a complete block of general translation. Although each of these kinases phosphorylates eIF2α on the same serine residue, they do so in response to differing stresses [56,95]. The PKR-like endoplasmic reticulum kinase (PERK) is mainly activated following ER stress, as part of the unfolded protein response (UPR) and has been shown in mice to be inhibited by AKT1-dependent phosphorylation [96,97,98]. The general control nonderepressable-2 (GCN2) is part of the nutrient sensing pathway and responds to amino acid starvation; lack of amino acids results in uncharged tRNAs, which stimulate GCN2 kinase activation [99]. Heme-regulated eukaryotic initiation factor-2-alpha kinase (HRI), which is expressed mainly in cells of erythroid lineage and the first of the eIF2α kinases to be identified, is activated in response to low heme concentrations [100]. The double-strand RNA-dependent kinase PKR, on the other hand, is activated in response to the most diverse types of stresses; among these are: viral infection, dsRNA, peroxidation, mitochondrial stress, DNA damage, ER stress, inflammatory cytokines, growth factor deprivation, and Toll-like receptor activation [101,102]. Together, these kinases form a network that can regulate translation initiation under a myriad of stress conditions [103,104,105]. Interestingly, while each of these kinases can be found in the cytoplasm, PKR is the only eIF2α kinase that is also present in the nucleolus and nucleoplasm [56,106].

**Figure 4:**
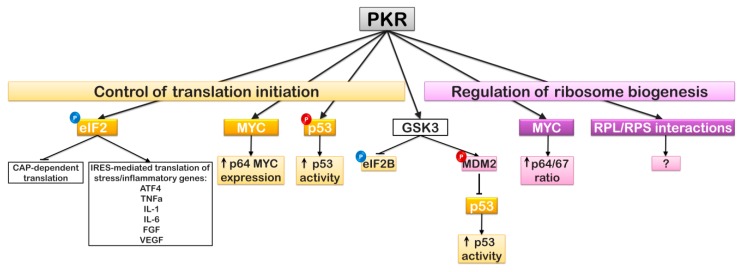
PKR regulates both translation initiation and ribosome biogenesis. The diverse points that PKR regulates in ribosome biogenesis and translation initiation are indicated. PKR regulates general CAP-dependent translation initiation and favors alternative translation initiation (ex., IRES) through the phosphorylation of eIFα or the modulation of GSK3α/β phosphorylation by phosphatases. Regulation of ribosome biogenesis is through PKR-mediated effects on p53 stability and MYC isoform expression. PKR likely also influences a number of additional proteins critical to ribosome biogenesis with which it interacts in the nucleus, including diverse ribosomal proteins. Phosphorylation (P) marked in red represent phosphorylations that enhance the activity of the recipient protein; phosphorylations in blue represent phosphorylations that are inhibitory to the recipient protein.

From prokaryotes to mammals, ribosome biogenesis and subsequent translation are highly regulated by the surrounding environment to limit energy expenditure under conditions that are unfavorable for growth, as well as limit the possibility of producing mutant proteins [107,108]. While phosphorylation of eIF2α was long thought to be strictly pro-apoptotic, this is not the case. Phosphorylation of eIF2α results in a shut-down of general CAP-dependent translation but, at the same time, it allows for efficient translation of upstream open reading frames (uORFs) in particular mRNAs that contain complex secondary structure at the 5’ end and an IRES element upstream [109,110,111]. Short-term inhibition of general translation through eIF2α phosphorylation establishes a pro-survival state by allowing for cellular repair and time for the cell to adjust following a particular stress [112]. If this stress cannot be resolved and general translation remains inhibited, the cell will likely die through apoptotic means; thus, a coordinated interaction between the eIF2α kinases and the AKT-mTOR-p70 S6K pathway must be present. In contrast, under other conditions, the phosphorylation of eIF2α has been shown to inhibit IRES-mediated translation [113]. These differences may be due, in part, to the presence of specific regulator proteins that differ between IRES elements and are, therefore, specific to the being mRNAs translated (Figure 4).

Many of the mRNAs translated under conditions where eIF2α is phosphorylated encode inflammatory cytokines such as TNFα, IL-1, FGF, VEGF, IL-6; or transcription factors, such as the cyclic AMP-dependent transcription factor, ATF4 [114,115,116,117,118,119]. Significantly, prolonged expression of each of these inflammatory mediators is associated with angiogenesis and tumor progression. ATF4 leads to increased synthesis of ATF3, which was shown to be a significant factor in low-risk MDS [120]. This aspect of chronic inflammation is noteworthy as ATF3 is considered a pro-apoptotic transcription factor. The anti-apoptotic effect mediated by PI3K-AKT-mTOR pathway inhibition in colorectal cancer was correlated to enhanced ATF3 expression [121]. In contrast, several lines of evidence argue against ATF3 being strictly pro-apoptotic. Ectopic overexpression of ATF3 in breast tissue led to breast carcinoma in mice [122]. Moreover, ATF3 was found to be highly overexpressed in classic Hodgkin’s lymphoma and contributes to the progression of this disease [123]. Whether constitutive overexpression of inflammatory cytokines like ATF4 is a secondary characteristic of these tumors, due to a ribosomal stress response that favors IRES-mediated translation or due to unrelated events has not been determined [56].

Phosphorylation of eIF2α also favors translation of the full-length isoforms of the CAAT-enhancer binding proteins, C/EBPα and C/EBPβ, which are both critical to hematopoietic development and differentiation [124]. Two isoforms of C/EBPα have been observed in cells, a full-length protein of approximately 40 kDa (p42) and a truncated protein of about 30 kDa (p30) [125]. While both p42 and p30 C/EBPα can interact with additional transcription factors, only p42 contains the entire transactivation domain [125]. Targeted deletion of C/EBPα in mice results in the inhibition of myeloid differentiation with subsequent blast accumulation [125,126]. Therefore, it is not surprising that C/EBPα has been found to be mutated or repressed in several hematologic malignancies where blast accumulation is a factor [125,127,128]. Interestingly, p30 C/EBPα can regulate the expression/accumulation of p42 C/EBPα through Ubc9 ubiquitin ligase-mediate ubiquitination of p42 C/EBPα, resulting in p42 degradation; thus, blocking differentiation in CD34+ hematopoietic stem cells, favoring self-renewal [56,129].

C/EBPβ is expressed as one of three forms: p38, p33, and p20, and in many ways is similar to C/EBPα [125]. But, whereas the loss of C/EBPα transcriptional activity is associated with tumorigenesis, loss of C/EBPβ is not [56,125,130,131].

Similarly, PKR is also known to regulate the expression of MYC. Diverse groups have demonstrated that PKR influences the expression of *c-myc* through the stimulation of the transcription factors nuclear factor (NF)-κB and signal transducers and activators of transcription (STAT) [132,133]. Blalock et al. demonstrated that pharmacological inhibition of PKR activity in an acute lymphoblastic leukemia cell line, where AKT activation was constitutively-active, resulted in enhanced expression of MYC. Not only did inhibition of PKR kinase activity enhance MYC expression, but it also influenced the isoform expression of MYC. Overexpression of PKR expression was shown to favor p64 MYC expression while siRNA-mediated knock-down of PKR favored p67 MYC expression [39]. The p64 isoform of MYC is initiated from a standard AUG start codon; in contrast, the p67 isoform is produced from a non-canonical CUG start codon and encodes an additional 15 amino acids at the amino terminus. Both p64 and p67 target E-box sites in MYC responsive promoters, but p67 can also target C/EBP elements, thus leading to the transcription of an additional set of responsive genes [39,40]. It has been suggested that the ratio p64/p67 dictates whether MYC expression favors growth and proliferation or stimulates the expression of pro-apoptotic factors, with p64 favoring proliferation and p67 favoring growth arrest [39,40]. This might suggest that the loss of PKR may stimulate a feedback control through MYC isoform expression to limit growth and proliferation of cells that do not have the necessary safeguards in place to monitor translation initiation (Figure 4).

The glycogen synthase kinase (GSK)-3β, which is inhibited by AKT under growth conditions but whose activity can be stimulated by the eIF2α kinases PKR and PERK through the action of phosphatases, phosphorylates diverse targets to inhibit growth and energy storage programs. Data from both hematological and neuronal models, in which AKT was constitutively-active, as a result of PTEN deletion, demonstrated that the inhibition of PKR in this genetic background, leads to increased inhibitory phosphorylation of GSK-3α/β on Ser21/9, and eventual cell cycle arrest [134,135]. Among the targets of GSK-3β is the eIF2B ε-subunit. eIF2B serves to exchange GTP for GDP bound to the eIF2 complex [136]. The eIF2B GTP exchange factor is composed of α, β, γ, δ, and ε subunits, of which the 82 kDa ε-subunit is the most critical to eIF2B regulation and is enzymatically responsible for the GDP to GTP exchange. The α-, β-, and δ-subunits associate with Ser51 phosphorylated eIF2α and inhibit eIF2Bε activity, locking eIF2B with eIF2 in the GDP-bound state while the γ-subunit, which is phosphorylated and regulated by casein kinase (CK)-II, promotes the activity of the ε-subunit. Phosphorylation of eIF2Bε on Ser535 by GSK-3β, following amino acid starvation, inhibits eIF2B activity [56,136,137]. For GSK-3β phosphorylation to occur, eIF2Bε must first be phosphorylated on Ser540 by one of the DYRK family kinases. The phosphorylation of these residue is thought to result in translation of a set of mRNAs that is different from those translated when only eIF2α is phosphorylated; thus, regulation of translation can give rise to proteins that are most efficiently translated under one of three (or more) different conditions [56,137,138]. Additional information on alternate translation can be found in the following reviews ([114,139,140,141]).

Stress signaling through PKR is also a critical component of p53 regulation. PKR directly phosphorylates Ser392 of p53, resulting in enhanced stability and transcriptional activity of p53. It has been observed that pharmacological inhibition of PKR in an active AKT background leads to the rapid degradation of p53 (unpublished results). Moreover, the activator of PKR, RAX/PACT, stimulates the sumoylation of p53 enhancing its activity, yet Baltzis et al. demonstrated that PKR and PERK lead to p53 degradation through GSK-3α/β-mediated phosphorylation and activation of MDM2. Under the conditions examined, GSK-3α/β resulted in enhanced ubiquitination of p53 and its subsequent nuclear export and degradation [142]. As GSK-3α/β was active in this study, it would stand to reason that a significant level AKT activity was not present in these cells under the conditions of the study. Thus, one might postulate that the fate of p53 relies on a balance between the eIF2α kinases (PKR and PERK) and AKT-mTOR activation (Figure 4). This would actually make sense as under stress conditions where AKT-mTOR still favored ribosome biogenesis and translation, PKR (or PERK in the case of ER stress) would result in p53 stabilization. In contrast, under conditions where a stress or insult occurs in the absence of a growth-promoting signal through AKT-mTOR, it may not be advantageous for the stress response through the interested eIF2α kinases to promote p53-dependent transcription.

Being associated with the 40S subunit, 60S subunit, 80S PIC, and polysomes, PKR is in the perfect position to respond to any cellular stress and regulate translation [143]. With the presence of PKR in the nucleus and the recent finding that it is associated with diverse proteins involved in ribosome biogenesis, it would not be surprising if PKR has other direct roles in the mechanisms responsible for protein synthesis other than simply that of eIF2α and p53 phosphorylation. A recent study by Blalock et al. reported that PKR isolated from nuclear lysates was associated with a number of ribosomal proteins of the 40S and 60S ribosomal subunits; the majority of which were found associated when PKR was active [39]. Treatment with a pharmacological inhibitor of PKR resulted in the dissociation of all but two of these RPs (RPS10 and RPS10-like) and the association of an additional four RPs (RPS19, RPS26, RPL23, and RPL36) (Figure 4). Interestingly, haplosufficiency of several of these proteins, RPS10, RPS19, and RPS26, is associated with Diamond–Blackfan Anemia (DBA; see below). Additional proteins found to associate with PKR were involved in nuclear protein import, rRNA synthesis/modification/processing, mRNA nuclear export, ribosome assembly, PIC assembly, MYC expression, and IRES-dependent translation. In all, approximately 60% of the proteins associated with PKR in the nucleus play a significant role in ribosome biogenesis and translation initiation (for a full list of the identified proteins, see Table 2). What was not determined in this study was which of the identified proteins was directly associated with PKR and/or a substrate of PKR; and if an associated protein did represent a substrate of PKR, what were the site(s) and significance of PKR-dependent phosphorylation of the protein [39]. 

## 7. RNA Editing/Splicing and Its Potential Role in Ribosome Biogenesis

RNA editing and alternative splicing of RNA are fundamental cellular processes that enhance the possibility of gene expression and increase protein diversity. Any impairment of these mechanisms is associated with the failure of normal cellular homeostasis resulting in disease [2,144]. The importance of RNA editing and/or splicing in coding RNAs (mRNAs) has become increasingly evident, but it may very well be the affect these processes have on non-coding RNAs (miRNAs, long non-coding RNAs, etc.) that is most critical to an organisms development and maintenance; and whose aberrant regulation may be responsible for diverse pathogenic conditions.

In coding mRNAs, alternative splicing can alter the amino acid sequence of the resulting protein, which can affect its function/activity or localization. It can also influence the translation efficiency and stability of the encoding mRNA and, thus, regulate protein expression post-transcriptionally (but pre-translationally) when it occurs in the 5′- and 3′-untranslated regions of the mRNA. Numerous interacting components of the spliceosome complex and associated heterogeneous nuclear RNPs (hnRNPs) are involved along with *cis*-acting elements in the primary transcript. This mechanism is finely regulated at developmental stages in different tissues, and an alteration in regulation of alternative splicing is now linked with several human diseases, including leukemia and pre-leukemic states such as MDS [2,145]. Splicing factor 3B subunit 1 (SF3B1), U2 small nuclear RNA auxiliary factor 1 (U2AF1), serine/arginine-rich splicing factor (SRSF2), and zinc finger CCCH-type, RNA binding motif and serine/arginine-rich 2 (ZRSR2) are splicing factors that carry recurrent somatic mutations in MDS and are components of the E/A splicing complex that coordinates 3′ splice site recognition during the early phase of pre-mRNA processing [2,146,147,148]. With regards to ribosomal proteins and regulators of ribosome biogenesis, the presence of alternatively spliced forms and their influence is just beginning to gain ground. Recent studies by Mei et al. and Rendleman et al. have demonstrated the presence of alternately spliced forms of p70 S6K1 and aminoacyl-tRNA synthetases, respectively. While the p70 S6K1 variant was found to be highly expressed in non-small cell lung cancer (NSCLC), the splice variants of aminoacyl-tRNA synthetases were observed under stress conditions and likely represent part of the cellular stress response [149,150]. Likewise, Mrvovà et al. reported the identification of diverse alternatively spliced forms of eIF4E family members in acute lymphoblastic leukemia cell lines [151]. These alternatively spliced mRNAs were shown to either contain different polyadenylation sites, affecting the stability of the mRNA, or encode diverse C-termini, potentially altering the function of the protein. Additionally, alternatively spliced transcripts of the DNA polymerase delta-interacting protein 3 (POLDIP3), a direct target of p70 S6K1, have been reported [152]. Most recently, the presence of alternatively spliced ribosomal proteins has been documented. Data across species agree that alternate forms of the ribosomal proteins due to alternative splicing are extremely rare; most alternative forms such as RPL10 versus RPL10-like (RPL10L) are instead due to tissue specific expression of one gene over another. Gupta and Warner reported that in the ENCODE database, of the 376 introns from RPs catalogued, only one was alternatively spliced [153]. Interestingly, this does not indicate that RP expression is not regulated by alternative splicing. Diverse groups have found that many RP mRNAs are alternatively spliced leading mRNAs termed “pseudogenes” that are targeted for non-sense mediated degradation. Plocik and Guthrie demonstrated in Drosophila, with RPS9, that the alternative spliced RPS9 mRNAs serve to regulate the accumulation and expression of RPS9, providing a clear example of how alternative splicing may influence RP expression without producing alternative protein isoforms [154]. Carlston et al. described in a recent case report, a 2-year-old boy with Diamond–Blackfan anemia resulting from a maternally inherited mutation that led to alternative splicing of RPL11 mRNA. The authors state that the resulting protein was susceptible to missense-mediated decay, accounting for the haploinsufficiency of RPL11 in this individual [155]. Two additional studies have suggested a role for the hnRNP complexes in RP alternative splicing. A study by Aviner et al. identified the hnRNP C, a protein–RNA complex involved in splicing, as being critical to the translation of mRNAs encoding ribosomal proteins and translation factors [156]. More importantly, Liu et al. recently reported that hnRNP K directly enhances the alternative splicing of mitochondrial RPL33 (MRPL33) producing MRPL33-L, which has increased tumor promoting potential and is associated with colorectal cancer [157]. Findings of frequent mutations in genes involved in RNA splicing in myelodysplasia and other diseases have resulted in the grouping of several pathologies as “spliceopathies” [158]. Furthermore, the activity of many splicing factors is regulated by phosphorylation through kinases such as AKT, CLKs, NEK2, PRP4, and TOP1. Alternative splicing may be a point of departure for the discovery of novel diagnostic and prognostic biomarkers as well as new therapeutic strategies to disease (for more detail, see [2]).

Other than nucleotide modifications to the RNA or the direct alteration and regulation of the splicing complex components, alternate splicing also involves sequence specific *cis*-elements that can be modified by a process known as “RNA editing”; thereby changing the splice acceptor/donor sites to generate alternatively spliced mRNAs. “RNA editing” is an important post-transcriptional mechanism, occurring in a wide range of organisms, which alters the primary RNA sequence through the insertion/deletion or modification of specific nucleotides. By far the most important mechanism is through the deamination of cytosine or adenosine residues. Three families of enzymes are primarily responsible for this type modification: The apolipoprotein B mRNA editing enzyme, catalytic polypeptide-like (APOBEC) family of cytidine deaminase members, APOBEC1, APOBEC2, and APOBEC4; the adenosine deaminase acting on tRNA family (ADAT1, ADAT2, and ADAT3), and the adenosine deaminase acting on double-strand RNA (dsRNA) family (ADAR1, ADAR2, and ADAR3).

**Figure 5:**
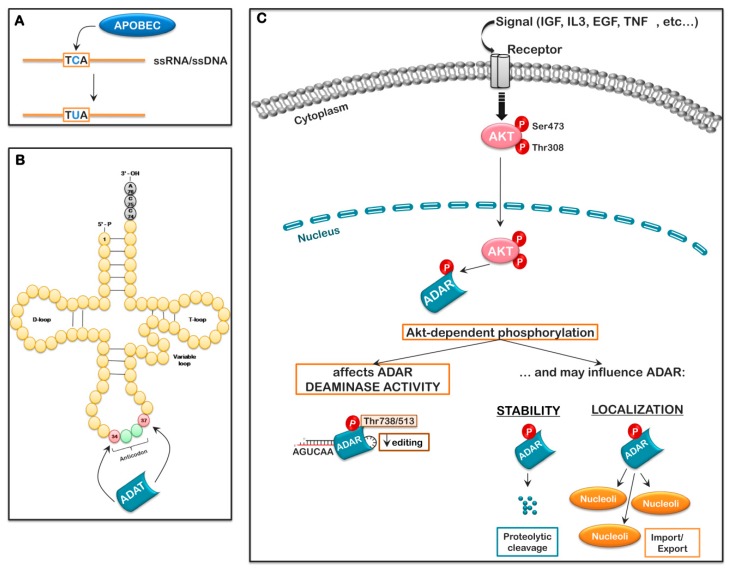
Adenosine deamination can modify diverse RNA species affecting ribosome biogenesis and translation. The APOBEC, ADAT, and ADAR families are capable of deaminating key adenosine (**A**) residues to inosine (I) in RNA resulting in alternative splicing, amino acid composition of proteins, siRNA target sequence recognition, snRNP activity, and RNA transport. In addition, each of these alterations can have pleotropic effects. (**A**) The APOBEC enzymes deaminate cytidine residues in ssRNA and ssDNA. (**B**) The ADAT family modifies adenosine residues in tRNA potentially altering translation initiation, elongation, or termination. (**C**) The ADAR family modifies adenosine residues in dsRNAs. Recently, AKT has been shown to phosphorylate and regulate ADAR editase activity.

The APOBEC family (APOBEC1, APOBEC2, APOBEC3A-H, APOBEC4, and activation-induced adenosine deaminase (AID)) is thought to be an innate immune-related gene cluster. Members of this family deaminate cytidine (C) or deoxycytidine (dC) to form uracil (U) or deoxyuracil (dU), respectively, in their targets, which vary greatly depending on the enzyme, tissue, and cellular localization [159]. The APOBEC3 members and AID target ssDNA. In contrast, APOBEC1, APOBEC2, and APOBEC4 are the only members of the cytidine deaminases to specifically target RNA. These enzymes work as homodimers or homotetramers, and their expression is tissue/organ specific: Small intestine (APOBEC1), heart and skeletal muscle (APOBEC2), or testes (APOBEC4) [159]. Although cytidine deaminases account for a minority of RNA editing events in the cell, mutations in several of these enzymes are associated with disease (Figure 5).

Adenosine deaminations to inosine (A-to-I editing), in contrast, account for the majority of RNA editing events in the cell. Inosine is interpreted by the cellular machinery as guanine, thus A-to-I RNA editing within mRNAs and ncRNAs (long ncRNA and miRNAs) increase the human RNA/protein landscape [160]. In pre-mRNAs, A-to-I editing can generate or destroy splice sites and alter codons, thus increasing proteome diversity. In addition, the modification of RNA transcripts at the 5′ and 3′ untranslated regions (UTRs) can alter translation and stability of the mRNA, respectively. Moreover, modifications of ncRNAs (miRNAs, long ncRNAs, snoRNAs, and scaRNAs) can alter their target specificity or function [160,161]. Recently, it has been reported that the A-to-I RNA editing frequency is massively increased from mouse to human [162]. In humans, most A-to-I RNA editing events (≥90%) occur within *Alu* inverted repeats, located preferentially in gene-rich regions. The majority of identified adenosine to inosine modifications in the cell have been linked to one of two families of adenosine deaminases: The ADAR family or the ADAT family. Ironically, both families consist of three members, with two members being active enzymes (ADAT-1 and -2; ADAR-1 and -2) and the third (ADAT3; ADAR3) demonstrated to be catalytically inactive.

The adenosine deaminase acting on tRNA (ADAT) family consists of ADAT1, ADAT2, and ADAT3 and is responsible for deamination within tRNAs, which represents a minor fraction of the A-to-I editing in the cell. The targets of ADAT1 and ADAT2 known to date are rather specific. ADAT1 specifically deaminates adenosine 37 in the anti-codon loop of tRNA-Ala. On the other hand, ADAT2 specifically deaminates adenosine 34 in a variety of tRNAs. To date, 7–8 different tRNAs containing I34 have been identified. ADAT3, which is highly homologous to ADAT2 over 120 amino acids of the deaminase motif, is believed to be catalytically inactive [163]. Editing of A-34-I is associated with the heterodimetric ADAT (hetADAT), which is composed of ADAT2/ADAT3; thus, ADAT3 regulates the activity of ADAT2. Cellular localization of the ADAT enzymes is strictly cytoplasmic [163,164]. The consequences of the modifications catalyzed by these enzymes have not been fully elucidated, but likely influence translation elongation under stress conditions, the possible use of altered initiation codons, premature termination of translation, or possibly allow codon slipping of the elongating ribosome to favor a shift in the reading frame [164,165,166]. The regulation of theses protein has not been thoroughly investigated (Figure 5).

In higher mammals, the most common type of RNA editing is mediated by the ADAR family within dsRNA regions of coding and non-coding primary transcripts. Like the ADATs, there are three highly conserved members of the ADAR family: ADAR1 (or DSRAD), ADAR2, and ADAR3. Both ADAR1 and ADAR2 are ubiquitously expressed (ADAR2 being most abundant in the brain), while ADAR3 expression is restricted to neural tissue, namely the brain [167]. Each of these enzymes contain dsRNA-binding domains (dsRBDs), three in ADAR1, and two in both ADAR2 and ADAR3, which allow them to bind/localize with their substrates, and a catalytic deaminase domain at the C-terminus. While ADAR1 and ADAR2 are active enzymes, ADAR3 is catalytically inactive [168]. Since these enzymes work as homo- or heterodimers, the presence of ADAR3 may serve to regulate ADAR1- and ADAR2-dependent editing or sequester substrates of ADAR1 and ADAR2. Unlike the ADAT deaminases, the localization of the ADARs is for the most part restricted to the nucleus, nucleoli, and nucleoplasm with the exception of ADAR1 [167]. Two main isoforms of ADAR1 are generated by alternate transcriptional initiation from exons 1A, 1B, or 1C. As exons 1B and 1C do not contain a start codon, translation of mRNAs beginning with these alternate exons initiates in exon 2, producing a protein of 103 kDa, known as ADAR1p110, which is mainly nuclear. In contrast, transcriptional initiation at exon 1A is strictly interferon-inducible, and, due to the presence of a translational start codon in exon 1A, the transcript produces a protein 295 amino acids longer (136 kDa), known as ADAR1p150, which can shuttle between the cytoplasm and nucleus [169,170]. On the other hand, two main forms of ADAR2 have been described, a result of alternative splicing, which are referred to as ADAR2 long (81 kDa) or the more enzymatically active ADAR2 short (ADAR2a; Δaa466-605; 77 kDa) [171].

Over the years, diverse targets of ADAR-mediated editing have been reported. Due to limited availability of high-throughput technology, many of the first RNA substrates identified were those in mRNAs that produce an identifiable change in the cDNA (as compared to the corresponding genomic sequences) and, in some cases, the amino acid sequence of the encoded protein. Advancements in technology and next generation sequencing have allowed for more large-scale analyses and identification of RNA editing sites within introns, *Alu* repeats, and non-coding RNAs, including many that play a role in ribosome biogenesis [168]. The function of many of these A-to-I modifications has not been examined. Of particular interest is the fact that while ADAR1p110 and ADAR2 are believed to localize to the nucleolus through interactions with the structural transcribed spacer regions of the rRNA, to date, in mammals, no known sites of A-to-I editing in rRNA have been reported [168,172]. The reason is likely two-fold: 1) rRNAs possess a very low adenosine content, thus limiting the available substrates for modification and 2) many current whole transcriptome studies use an rRNA depletion step to reduce background noise and non-specificity. In contrast, Eifler et al. reported that ADAR2 was able to modify the 25S rRNA as well as the splicing complex RNAs U1 snRNA and U2 snRNA in yeast [161]. Whether the ADARs directly modify rRNAs or not, their presence in the nucleolus and nucleoplasm likely has significant consequences on ribosome biogenesis through recruitment and/or modification of secondary proteins and RNAs necessary for rRNA synthesis and maturation.

As stated, little information exists on the regulation of these enzymes beyond their expression and the fact that multiple splice variants are reported. Multiple studies have identified post-translational modifications on numerous residues in ADAR1 and ADAR2 in vivo (https://www.phosphosite.org/proteinAction.action?id=10051&showAllSites=true and https://www.phosphosite.org/proteinAction.action?id=5859&showAllSites=true), and several recent publications have demonstrated the importance of phosphorylation to ADAR activity. Sakurai et al. demonstrated that stress-induced phosphorylation of ADAR1p110 in the disorganized region between dsRNA-binding domain III and the editase domain by the MKK6-p38-MSK MAPK pathway resulted in the association of Exportin-5 with ADAR1p110 and the export of ADAR1p110 from the nucleus to the cytosol, where it blocks Staufen 1-mediated decay, promoting apoptosis [173]. Likewise, Shelton et al. demonstrated that ADAR2 was phosphorylated at two sites between the dsRNA-binding domains, Ser211 and Ser216, by PKCξ. Phosphorylation at these sites was demonstrated to regulate ADAR2 RNA editing activity toward miR-200, and the subsequent secretion of this miRNA [174]. Bavelloni et al. recently reported that both ADAR1p110 and ADAR2 are substrates for the AKT kinase family. Inhibition AKT with either the allosteric inhibitor, MK2206, or the ATP binding site inhibitor, AZD5363, resulted in enhanced ADAR1- and ADAR2-dependent editing of known substrates in treated U-87MG cells. AKT1 was found to primarily phosphorylate S738 in ADAR1p110 and S553 in ADAR2, within the catalytic domain. Expression of either phosphomimic mutant, ADAR1p110-S738D or ADAR2-S553D, resulted in reduced ADAR1- and ADAR2-dependent editing [74]. A full analysis of the effects of this regulation on global ADAR-dependent editing is still needed. Thus, not only can downstream signaling of the PI3K-AKT pathway influence ribosome biogenesis through mTOR and p70 S6K, it potentially has a significant role in regulating global regulators of RNA metabolism/processing (Figure 5).

Additionally, ADAR1 has a strict relation with PKR in stress granules. Stress granules are cytoplasmic aggregates of stalled 40S ribosomal subunit-containing translation initiation complexes linked to eIF2α Ser51 phosphorylation and are a hallmark of negative-strand RNA virus infection. ADAR1p150 was shown to co-localize with PKR in stress granules in the cytoplasm and inhibit PKR activity. George et al. demonstrated that in cells lacking ADAR1 (ADAR1-/-), treatment with IFN lead to increased eIF2α Ser51 phosphorylation and PKR-dependent stress granule formation [175]. Interestingly, similar to other components of ribosome biogenesis, it has been shown that ADAR1 expression and editase activity are required for normal erythropoiesis, as cells deficient in ADAR1 undergo enhanced apoptosis. [176] In the nucleus, PKR is known to directly bind ADAR1p110, but the significance of this interaction has not yet been elucidated [39]. Seeing that PKR and AKT often play a tug-of-war, it is possible that nuclear PKR activity is also regulated by ADAR1 or that ADAR1p110 may represent a substrate for active nuclear PKR. Given the biological relevance of RNA editing in mammals, it has been postulated that its deregulation could be linked to a variety of human disorders.

## 8. Ribosome Biogenesis and Disease (Ribosomopathies)

Several pathologies have their etiology founded in altered ribosome biogenesis and result from both acquired as well as hereditary mutations. Interestingly, while hereditary germline alterations are present in every nucleated cell of the organism, the associated pathologies strictly affect tissues and cells that have a high demand for protein synthesis or proliferation/cell turnover. Almost all these pathologies exhibit a large hematological component and patients with ribosomopathies almost always present with hematopoietic abnormalities, due to bone marrow failure. Erythroid progenitors are particularly sensitive due to the high demand for globin synthesis and rapid cell turnover. Thus, it is no wonder a number of the ribosomopathies are also termed bone marrow failure disorders (BMFDs). Patients with ribosomopathies/BMFDs often have clinical signs of a chronic, overactive innate immune/inflammatory response with elevated levels of circulating inflammatory mediators, such as TNFα, IL-1α, and IFNγ, which suppress the growth of progenitors and stimulate apoptosis in the bone marrow and other hypersensitive tissues [2,177,178]. Interestingly, while ribosomopathies appear to have a pro-apoptotic effect on the affected tissues, individuals who suffer from these pathologies have an increased incidence of developing cancers. One hypothesis is linked to the association between chronic inflammation and cancer. Ribosomopathies may represent the classic case of a chronic inflammatory state affecting tissues with an elevated necessity for protein synthesis and cell turnover. Prolonged ribosomal stress, like chronic inflammation, may place a selective pressure on the affected cells. Progenitor cells that are able to escape this selective pressure and proliferate are cells that have, over time, acquired random gain of function mutations, leaving them with characteristics of cancer stem cells [2,56,179].

### 8.1. Acquired Ribosomopathies: 5q-Syndrome

As far as acquired ribosomopathies go, only one pathology currently stands-out, 5q- myelodysplastic syndrome (MDS; 5q- syndrome). Myelodysplastic syndromes are a heterogeneous group of hematological malignancies that result in cytopenias in one or more of the hematologic lineages, with or without cytogenetic abnormalities. *De novo* MDS typically occurs in later life (>60 years of age) and is, thus, considered an age-related disorder. In contrast, MDS can also arise from the progression of other hematological malignancies, including other BMFDs. Moreover, therapy-related MDS (tMDS) is known to occur in individuals previously treated with chemotherapeutic agents for diverse types of cancer [2,180].

MDS is categorized by the International Prognostic Scoring System (IPSS) as low, intermediate-1, intermediate-2, and high, based on the risk of the disease progressing to acute leukemia. In low-risk disease, there is a propensity for hematologic stem cells and progenitors to undergo apoptosis with few blasts observed in the peripheral blood. This is gradually replaced during disease progression from low- to high-risk, where there is hypercellularity of the bone marrow and the appearance of blasts in the periphery [2,180,181].

Diverse groups have demonstrated a role for stress/inflammatory signaling during progression of MDS to AML [56,134,182]. More recently, significant alterations in DNA methylation, chromatin modification, transcriptional regulation, DNA repair, signal transduction, sister chromatid cohesion and RNA splicing, and ribosome biogenesis associated genes have been observed. Certain mutations are specific to particular subtypes of myelodysplasia [2,148,183,184]. In the case of 5q- syndrome, a deletion of the long arm of chromosome 5, which encodes *rps14*, results in a haploinsufficiency of RPS14 [48]. Loss of RPS14 results in defective ribosome biogenesis and translation, the stimulation of p53 transcriptional activation, cell cycle arrest, and enhanced apoptosis especially in erythroid progenitors, resulting in anemia [185]. To date, 5q- syndrome is the only myelodysplastic disease to be labeled a ribosomopathy. This is not to say that other types of MDS or other acquired BMFDs might not, in fact, be ribosomopathies, but currently, data does not exist to conclusively suggest that these other acquired BMFDs result from an altered ribosome component.

### 8.2. Acquired Ribosomopathies (Potential): T-Cell Acute Lympoblastic Leukemia

A number of cancers that are not currently classified as ribosomopathies have been found to contain mutations in ribosomal proteins, which result in defective or altered ribosome synthesis; these include glioma, colorectal cancers, chronic lymphocytic leukemia (CLL), and T-cell acute lymphoblastic leukemia (T-ALL) [186]. In the case of T-ALL, mutations in RPL5, RPL10, and RPL11 have been identified, with mutations in RPL5 or RPL10 being found in about 10% of pediatric T-ALL patients [187]. One of the original driver mutations observed in T-ALL was that of the NOTCH1 receptor. Activating NOTCH1 mutations are present in more than 50% of T-ALL cases. Pioneering work by Palomero et al. identified the loss of PTEN function and subsequent PI3K-AKT-mTOR pathway activation as critical to mutant NOTCH1 effects [188]. Suppression of PTEN function has been demonstrated to occur by both genetic and non-genetic mechanisms. Anywhere from 11%–27% of pediatric T-ALL patients show deletions, insertion, or point mutations in the PTEN gene, accounting for reduced activity [189]. Reduced PTEN activity has also been linked to alternate splicing of PTEN mRNA, phosphorylation of PTEN by casein kinase 2 (CK2), and miRNA targeted decay of the transcript [189]. In addition, aberrant NOTCH1 activity results in the activation of the downstream transcription factor target, hairy and enhancer of split-1 (HES1), which suppress PTEN expression [188,189]. Thus, aberrant NOTCH1 signaling and PTEN suppression are critical to T-ALL clonality and maintenance. What remains elusive though is, do these alterations favor mutations in the observed ribosomal proteins or do mutations in the observed ribosomal proteins favor the alterations of NOTCH1 and PTEN. A case could be made for either.

It is evident in most ribosomopathies that defective ribosome biogenesis presents a selective pressure on the cell that over time leads to the development of a population of cells that have acquired the potential to overcome this obstacle, giving these cells a selective advantage. Two recent publications could make the argument that this is at play in T-ALL. Chronic inflammation is known to play a role in diverse metabolic pathologies. Villegas et al. found that the pro-inflammatory enzymes, nitric oxide synthase (NOS) and lipoxygenase (LOX), stimulated NOTCH-PI3K/AKT oncogenesis and that inhibition of these pro-inflammatory enzymes was able to suppress NOTCH-PI3K/AKT signaling, resulting in leukemic cell death [190]. These data link NOTCH-PI3K/AKT signaling directly to inflammation. Interestingly, while oncogenic transformation can induce inflammation, one of the definitive side-effects of altered ribosome biogenesis observed in ribosomopathies is a pro-inflammatory phenotype. Even more interesting was the finding by Grzes et al. that primary T-ALL cells have an increased capacity for leucine uptake and transport [191]. As stated previously, the uptake of leucine can stimulate mTORC1 activity at the lysosome, and treatment of patients with certain hereditary ribosomopathies with L-leucine is able to alleviate the pro-apoptotic phenotype and resulting anemia. Interestingly, the authors found that PTEN deletion alone could not result in L-leucine uptake; NOTCH1 signaling was required. NOTCH1 signaling resulted in enhance expression of the sodium-independent solute carrier family 7, member 5 transport protein, which is involved in leucine uptake and transport [191]. Thus, one might argue that alterations in ribosome biogenesis may favor NOTCH1 mutations. Data from Sulima et al. might also support this scenario [192]. Their data has demonstrated that the most common RPL10 mutation observed in pediatric T-ALL, Arg98Ser, can induce a ribosome deficiency that results in a hypoproliferative state. Over time, these ribo-deficient cells acquire the ability to produce sufficient ribosomes and proliferate normally. While these cells can proliferate normally in the long-run, they produce defective ribosomes that result in both genomic and mRNA transcript instability, alterations often observed in advanced cancers, including T-ALL [192].

### 8.3. Hereditary Ribosomopathies

Hereditary ribosomopathies include: Diamond–Blackfan anemia (DBA), Shwachman–Diamond syndrome (SDS), Dyskeratosis congenital (DC), and Treacher–Collins syndrome (TCS). All involve the presence of an inherited mutation in one particular gene (SDS, TCS) or one member of a set of genes involved in a common cellular process (DBA, DC), which is ultimately responsible for the observed disease phenotype [178]. In ribosomopathies, where a mutation in more than one gene can cause the same disease, the pattern of inheritance and the penetrance of the disease phenotype may vary greatly. The involvement of particular tissues also depends at what point ribosome biogenesis and/translation is affected [2,177].

### 8.4. Diamond–Blackfan Anemia (DBA)

Diamond–Blackfan Anemia (DBA) is a rare congenital bone marrow failure disorder often noted within the first year or two after birth. DBA is characterized by a profound anemia, with growth retardation, cranialfacial abnormalities, and defects in the heart and urinary system being observe in up to 50% of patients. Affected individuals have an increased incidence of developing MDS and AML, as well as some other forms of cancer [2,178,193]. DBA results from altered ribosomal RNA (rRNA) processing which, in turn, affects ribosome biogenesis of the 40S and 60S ribosomal subunits and alters mRNA processing/transport and translation; thus, having pleiotropic effects on cellular growth and survival [2,193].

DBA, to date, has been associated with diverse mutations in the following ribosomal proteins: RPS7, RPS10, RPS17, RPS19, RPS24, RPS26, RPS27, RPS29, RPL5, RPL11, RPL26, RPL27, RPL35A, and the deletion of RPL15. Other novel mutations have been reported in RPL3L, RPL6, RPL7L1T, RPL8, RPL13, RPL14, RPL18A, and RPL31. Approximately 50% of DBA patients contain mutations in RPS7, RPS10, RPS17, RPS19, RPS24, RPS26, RPS29, RPL5, RPL11, RPL26, RPL35A, or GATA. Of these, mutations in RPS19 alone account for ~25% of these patients [2,194]. The other 50% of cases have, as of yet, no identified causative mutation, but are almost assuredly mutations associated with pre-rRNA processing and ribosome assembly.

There is some evidence that altered ribosome biogenesis may induce an inflammatory component. In patients bearing a RPS19 deficiency, the levels of GATA1 were found to be reduced in the erythroid progenitor population while p53 and TNFα expression were increased in the non-erythroid progenitors [26,193]. Inhibition of TNFα in a zebrafish model of RPS19-deficiency was able to rescue the observed anemia, suggesting TNFα expression has a significant role in the observed phenotype [2,26]. Similarly, stimulation of protein translation through the use of supplemental leucine has been reported to rescue the observed anemia [48].

### 8.5. Shwachman–Diamond Syndrome (SDS)

Shwachman–Diamond syndrome is an autosomal recessive disorder which initially manifests as an exocrine pancreatic dysfunction, but then results in bone marrow failure and skeletal abnormalities. Approximately 20% of SDS patients will progress to MDS while another 25% will develop AML [2,178]. In almost all cases (~90%), patients carry a mutation in the Shwachman–Blackfan-Diamond syndrome (SBDS) gene. The gene encoding SBDS is located on chromosome 7q11 and is immediately adjacent to its pseudogene, SBDSP, which is 97% identical to SBDS but contains deletions and nucleotide changes that prevent the expression of a functional protein. Interestingly, it is a recombination with this pseudogene that results in SBDS mutations in 75% of patients [2,23]. The SBDS gene encodes a 250 amino acid (29 kDa) protein that contains no known structural domains. SBDS is expressed ubiquitously in tissues and is localized throughout the cell with a particular preference for the nucleolus where ribosome biogenesis occurs. Mouse knock-out models and the fact that a common early truncation mutation at nucleotide 183 (TA > CT) is observed only in individuals heterozygous for the defect, suggest that loss of SBDS is embryonic lethal [2,23].

As is the case for most bone marrow failure disorders, the number of CD34+ hematopoietic cells in the bone marrow is reduced, and these cells show a reduced proliferative and colony forming capacity when compared to normal CD34+ hematopoietic cells. In addition, elevated p53 expression and increased apoptosis are observed in the bone marrow of SDS patients [2,23].

SBDS associates with Nip7, a 60S ribosomal subunit assembly factor and eIF6 associated with the 60S ribosome. In the assembling 60S ribosomal subunit, eIF6 serves in the proper maturation of the subunit and to inhibit the premature assembly of the 40S and 60S subunits to form the 80S ribosome. Mutational rescue data suggest that SBDS serves to disassociate eIF6 from the 60S subunit once it reaches the cytoplasm. This occurs by the SDBS-dependent recruitment of the cytoplasmic GTPase EFL1 to the eIF6-60S subunit complex. EFL1 promotes the dissociation of eIF6 from the 60S subunit, allowing for 80S assembly [2,23,24]. Thus, during ribosome biogenesis, reduced expression of SBDS hampers the dissociation of eIF6. In addition to the 60S ribosomal subunit, SBDS has also been found in association with 28S rRNA and NPM1, so that, depending on the mutation in this gene, the observed phenotype may vary greatly [2,23]. SBDS has also been shown to associate with microtubules during mitosis. SBS patients often show increased incidence of mitotic abnormalities, with multi-polar spindles and centrosomal amplifications, but the general consensus is that disease-causing mutations in SBDS result in altered 60S ribosome biogenesis and an enhanced sensitivity to stress [2,23].

### 8.6. Dyskeratosis Congenita (DC)

Dyskeratosis congenital (DC) is a highly rare multi-system progressive bone marrow failure disorder that can either present an autosomal dominant, autosomal recessive, or X-linked inheritance pattern base or the causative mutation. Its penetrance can vary widely extending from barely detectible to severe as in the case of Hoyeraal Hreidarson syndrome [2,178,195]. Dyskeratosis presents with a triad of symptoms that include reticulated skin hyperpigmentation, nail dystrophy, and mucosal leukoplakia and results in premature death resulting from bone marrow failure, respiratory dysfunction, or malignancy.

While dyskeratin (*DKC1*), which encodes for a protein involved both in the small nucleolar ribonucleoprotein (H/ACA snoRNP) and in the telomere complexes, and whose mutation is responsible for the X-linked form of DC, is by far the better studied; other proteins involved in the H/ACA snoRNP complex (*NOP10* and *NHP2*), the telomerase ribonucleoprotein complex shelterin (*TINF2*); *TERT*, the telomerase reverse transcriptase; *TERC*, which encodes the RNA component of TERT and whose mutation is responsible for the autosomal dominant form of DC; *WRAP53*, which delivers TERC to the telomerase as well as binds Cajal body RNAs (scaRNAs) and regulates p53 mRNA levels post-transcriptionally; *RTEL1*, which is involved in telomere elongation; and *CTC1*, a subunit of the CTC complex which terminates TERT activity and recruits DNA polymerase for complement strand synthesis, are also implicated [2,178,195]. All these proteins are involved in telomere maintenance, while several are also directly involved in small ribonuclear RNA processing, resulting in pseudouridylation of the snRNAs. Consensus seems to hold that the underlying cause of DC is the effect these mutations have on the telomeres and not the ribosomal effects per se. Although it should be stated that the transcription of the rDNA genes is closely related to genome stability, alterations in the telomerase complex would be expected to suppress rRNA transcription, while alterations in the H/ACA snoRNPs would be expected to influence rRNA processing [2,196,197]. As might be expected, tissues with the greatest proliferative/turnover rates are the most affected by DC.

### 8.7. Treacher–Collins Syndrome (TCS)

Treacher–Collins syndrome is an autosomal dominant disorder arising from an alteration in the *tcof1* gene, which encodes the Treacle protein. A deletion of 5 bps in exon 24 accounts for about 20% of the cases. Individuals have midface hypoplasia, an underdeveloped outer ear structure, inner ear abnormalities, and developmental brain defects. Treacle co-localizes with UBF and RNA pol I to stimulate the transcription of the 47S pre-rRNA. Mouse models of TCS have demonstrated the upregulation of p53 and pronounced apoptosis of neural crest cells [177].

## 9. Conclusions

The cell has gone to great lengths to place multiple checks or ribosome biogenesis and translation initiation to avoid useless energy expenditure under unfavorable conditions (both intracellular and extracellular). Technological advances in next-generation sequencing and cryo-EM have begun to open the door on many aspects of ribosome biogenesis. Studies have demonstrated the order of RP incorporation into the maturing ribosome subunits and where in the cell these events take place. DNA/RNA sequencing studies have also begun to identify mutations in the many proteins, mRNAs, and ncRNAs related to ribosome biogenesis and translation initiation that are associated with ribosomopathies. It is well evident that the notion of one kinase phosphorylating one substrate to regulate a particular process is past. Omics technologies have revealed a variety of modifications that take place on the same protein, ranging from sumoylations, ubiquitinations, acetylations, and the most wide-spread post-translational modification, phosphorylation. Certainly, some of these modifications are determinant in the activity of the protein, and many key modifications are already known; but, the purpose/function of the majority of these modifications and their consequences are unknown. This is no more evident than in the processes of ribosome biogenesis and translation initiation. While research to date has given a basic understanding of the processes, omics technology has revealed how complex the regulation of these processes really is. The combination of modifications and the possible outcomes is infinite along with the possibility of disease-causing alterations. The evolutionary relevance of these modifications allows the cell the possibility to respond to an infinite number of environmental stresses, much the same as the immune system is designed to recognize an infinite number of antigens. This review has touched upon two key regulation pathways, PI3K-AKT-mTORC1 and PKR in ribosome biogenesis and translation, and how these pathways interact with transcription factors (MYC and p53) and splicing/RNA editing enzymes. From the data, it is evident that our knowledge of both these pathways is only touching the tip of the iceberg. Combining the information gleaned from omics studies with biochemical/molecular studies to identify the function of these modifications will be paramount to our complete understanding of these processes and the future development of therapies to a variety of diseases, many with causes that at the present remain unknown.

## Figures and Tables

**Table 1:** Identified by tandem affinity purification-mass spectrometry (AP-MS/MS) using anti-AKT/p70 S6K phosphosubstrate antibodies.

Acc #	Gene Name	Name	Function	AKT pSites	Known Sites	AKT/p70 S6K Sub
P55265	*ADAR*	Double-stranded RNA-specific adenosine deaminase (NB4)	Catalyzes the hydrolytic deamination of multiple adenosines to inosines in RNA. This can result in diverse effects as a consequence of RNA modification.	(5/7)	Y (1/1)	X
Q86V81	*ALYREF*	THO complex subunit 4	Export adapter protein; functions in the export of spliced and unspliced mRNAs from the nucleus and mRNA processing.	(2/2)	Y (2/2)	X
Q9UIG0	*BAZ1B*	Tyrosine-protein kinase BAZ1B	Chromatin remodeling factor; involved in the promoting RNA polymerase (pol I, pol II, and pol III) activity.	(11/12)	Y (3/4)	
P17844	*DDX5*	Probable ATP-dependent RNA helicase DDX5	RNA helicase; involved in alternate pre-mRNA splicing;	(1/4)	Y (1/3)	
Q92841	*DDX17*	Probable ATP-dependent RNA helicase DDX17	RNA helicase; involved in RNA splicing, alternative RNA splicing, alteration of RNA secondary structure; involved in rRNA and miRNA processing; a transcriptional coactivator.	(6/7)	Y (3/3)	
Q9NR30	*DDX21*	Nucleolar RNA helicase 2	RNA helicase that senses the status of RNA pol I and RNA pol II activity; binds rRNAs, snoRNAs, and mRNAs; influences RNA pol II transcription; binds dsRNA and acts as a sensor for cytoplasmic dsRNA; activates inflammatory cascade.	(3/4)	N	
Q08211	*DHX9*	ATP-dependent RNA helicase A	RNA-DNA helicase with role in DNA replication, RNA transcription, translation, and RNA silencing; hnRNP actin binding. Transcriptional activator; mediates MYC mRNA stability: Interacts with RELA, IGFBP1, CREB-BP. Involved in viral infection and inflammasome activation; known substrate for EIF2AK2 (PKR).	(7/9)	N	
O43143	*DHX15*	Pre-mRNA-splicing factor ATP-dependent RNA helicase DHX15	RNA helicase; pre-mRNA splicing factor involved in the disassembly of the spliceosome.	(3/4)	Y (1/1)	
Q99848	*EBNA1BP2*	Probable rRNA-processing protein EBP2	Required for the processing of the 27S pre-rRNA; interacts with Ebstein–Barr virus (EBV) EBNA1 protein; required for stable EBV episome segregation	(1/1)	N	
P68104	*EEF1A1*	Elongation factor 1-alpha 1	Promotes the GTP-dependent binding of the aminoacetyl-tRNA to the A site of the ribosome.	(1/1)	N	
P05198	*EIF2S1*	Eukaryotic translation initiation factor 2 subunit 1	Alpha subunit of the eIF2 translation initiation factor; forms the ternary complex with GTP and the initiating tRNA. GTP hydrolysis catalyzes the formation of the 80S initiation complex.	(1/1)	Y (1/1)	
P20042	*EIF2S2*	Eukaryotic translation initiation factor 2 subunit 2	Beta subunit of the eIF2 translation initiation factor; forms the ternary complex with GTP and the initiating tRNA. GTP hydrolysis catalyzes the formation of the 80S initiation complex.	(2/2)	Y (1/1)	
O15371	*EIF3D*	Eukaryotic translation initiation factor 3 subunit D	mRNA CAP-binding component of the eIF3 complex; eIF3 is responsible for the recruitment of other initiation factors to form the 43S PIC; stimulates recruitment of mRNA to the 43S PIC and codon scanning to localize the initiator AUG.	(2/2)	Y (1/1)	
Q99613	*EIF3C*	Eukaryotic translation initiation factor 3 subunit C	Component of the eIF3 complex; eIF3 is responsible for the recruitment of other initiation factors to form the 43S PIC; stimulates recruitment of mRNA to the 43S PIC and codon scanning to localize the initiator AUG.	(1/2)	N	
O60841	*EIF5B*	Eukaryotic translation initiation factor 5B	Translation GTPase that catalyzes the assembly of the 80S translation initiation complex.	(4/4)	Y (2/2)	
Q15717	*ELAVL1*	ELAV-like protein 1	Ribonucleoprotein complex; involved in 3′ UTR AU-rich element (ARE) dependent MYC, FOS, and IL3 stabilization; binds p53 mRNA to facilitate its export from the nucleus.	(1/2)	Y (1/1)	
Q8IY81	*FTSJ3*	Pre-rRNA 2’-*O*-ribose RNA methyltransferase FTSJ3	RNA 2’-O-methyltransferase involved in early processing of 18S rRNA and formation of the 40S ribosomal subunit; maturation of the 5.8S rRNA.	(4/4)	Y (1/1)	
P35637	*FUS*	RNA-binding protein FUS	DNA/RNA -binding protein that influences transcription, RNA splicing, RNA transport and DNA damage repair.	(2/2)	Y (2/2)	
P09651	*HNRNPA1*	Heterogeneous nuclear ribonucleoprotein A1 (NB4)	Involved in pre-mRNA packaging into hnRNP; affects nuclear-cytoplasmic transport of polyA RNA; affects splicing.	(2/2)	Y (2/2)	X
P52597	*HNRNPF*	Heterogeneous nuclear ribonucleoprotein F	A component of the hnRNP complexes; involved in pre-mRNA processing.	(3/4)	Y (3/3)	
Q00839	*HNRNPU*	Heterogeneous nuclear ribonucleoprotein U	DNA/RNA-binding protein involved in RNA splicing, alternative splicing, and stability; influences chromatin structure and suppresses RNA pol II-dependent transcription.	(2/4) (m)	Y (2/2) (m)	
Q12905	*ILF2*	lnterleukin enhancer-binding factor 2	Functions as a heterodimer with ILF3 to regulate transcription of IL-2.	(2/2)	Y (1/1)	
Q07666	*KHDRBS1*	KH domain-containing, RNA-binding, signal transduction-associated protein 1	RNA-binding protein that regulates nuclear-cytoplasmic export and alternative splicing of mRNA.	(3/3)	Y (1/1)	
Q9NX58	*LYAR*	Cell growth-regulating nucleolar protein	Acts as a transcriptional regulator; functions in the processing of 47S rRNA to 18S and 28S rRNAs; part of the 90S, 60S, and 40S RNP complexes, but not polysomes; prevents nucleolin self cleavage.	(2/2)	Y (2/2)	
P43243	*MATR3*	Matrin-3	May function in the nuclear retention of defective RNAs; involved in the activation of the innate immune response.	(8/9)	Y (8/8)	
Q9BQG0	*MYBBP1A*	Myb-binding protein 1A	DNA-binding protein that may activate or repress transcription; has a role in ribosome biogenesis.	(7/10)	Y (3/4)	
Q9H0A0	*NAT10*	RNA cytidine acetyltransferase	RNA cytidine acetyltransferase; modifies mRNA, 18S rRNA, and tRNA; enhances translation efficiency; may acetylate lysine in some proteins such as p53.	(4/6)	N	
P19338	*NCL*	Nucleolin (NB4)	RNA-binding protein that influence RNA pol I and pol II transcription; plays a role in ribosome assembly	(2/2)	Y (2/2)	
Q15233	*NONO*	Non-POU domain-containing octamer-binding protein	DNA/RNA-binding protein involved in pre-mRNA splicing; plays a role in nuclear retention of defective RNAs; involved in DNA double-strand break repair; serve a role in ILF3 phosphorylation and innate immune response activation.	(1/2)	N	
P46087	*NOP2*	Probable 28S rRNA (cytosine (4447)-C(5))-methyltransferase NOP2	S-adenosyl-L-methionine-dependent methyltransferase that specifically methylates the cytosine 4447 in 28S rRNA; involved in the assembly of the 60S ribosomal subunit.	(6/7)	Y (4/5)	
O00567	*NOP56*	Nucleolar protein 56	Core component of box C/D small nucleolar ribonucleoprotein (snoRNP) particles. Required for the biogenesis of box C/D snoRNAs; involved in the processing and maturation of the 60S ribosomal subunit.	(4/5)	Y (3/4)	
P06748	*NPM1*	Nucleophosmin (NB4)	Involved in cellular division, ribosome biogenesis, and ribosomal export; regulates p53 and p14^ARF^; enhances MYC transcriptional activity; involved in assembly and export of the 40S and 60S ribosomal subunits; negatively regulates EIF2AK2 (PKR).	(1/1)	Y (1/1)	
P09874	*PARP1*	Poly [ADP-ribose] polymerase 1	DNA ribosyltransferase; promotes RNA pol II-dependent transcription; involved in DNA repair.	(2/2)	N	
Q6P2Q9	*PRPF8*	Pre-mRNA-processing-splicing factor 8	RNA-binding protein that associates with both 5’ and 3’ splice sites to position the U2, U5, and U6 for spliceosome formation.	(5/5)	Y (2/2)	
Q9UMS4	*PRPF19*	Pre-mRNA-processing factor 19	Ubiquitin protein ligase involved in pre-mRNA spliceosome assembly and DNA repair.	(3/3)	Y (1/1)	X
Q09028	*RBBP4*	Histone-binding protein RBBP4	Component of the chromatin assembly factor 1 (CAF-1) complex, which is required for chromatin assembly following DNA replication and DNA repair; the core histone deacetylase (HDAC) complex, which promotes histone deacetylation and consequent transcriptional repression; the nucleosome remodeling and histone deacetylase complex (the NuRD complex), which promotes transcriptional repression by histone deacetylation and nucleosome remodeling; the PRC2/EED-EZH2 complex, which promotes repression of homeotic genes during development; and the NURF (nucleosome remodeling factor) complex.	(1/10) (m)	N (m)	
Q96PK6	*RBM14*	RNA-binding protein 14	Acts a transcriptional coactivator (isoform 1) or repressor (isoform 2); aids in the activation of the innate immune response through ILF3 activation.	(2/2)	Y (2/2)	
Q14498	*RBM39*	RNA-binding protein 39	Transcriptional coactivator involved in RNA processing and splicing.	(16/18)	Y (7/8)	
P38159	*RBMX*	RNA-binding motif protein, X chromosome	RNA-binding protein that regulates pre- and post-transcriptional processes; involved in RNA pol II transcription; involved in mRNA splicing and alternative splice site selection.	(5/9)	Y (5/9)	
P39023	*RPL3*	60S ribosomal protein L3 (NB4)	Component of the large ribosomal subunit; binds 5S rRNA.	(3/3)	Y (1/3)	
P36578	*RPL4*	60S ribosomal protein L4 (NB4)	Structural component of the 60S ribosomal subunit.	(1/1)	Y (1/1)	
P62917	*RPL8*	60S ribosomal protein L8	Structural component of the 60S ribosomal subunit; binds rRNA.	(1/2) (m)	Y (0/1) (m)	
P26373	*RPL13*	60S ribosomal protein L13 (NB4)	Structural component of the 60S ribosomal subunit.	(4/5)	Y (3/4)	
P40429	*RPL13A*	60S ribosomal protein L13a (NB4)	Associated with the ribosome but is not a required component; associates with 3’-UTR inflammatory mRNAs; interacts with eIF4G near the eIF3 binding site to prevent 43S ribosomal complex assembly.	(1/1)	N	
P84098	*RPL19*	60S ribosomal protein L19	Structural component of the 60S ribosomal subunit; 5.8S rRNA binding.	(2/2)	Y (2/2)	
P23396	*RPS3*	40S ribosomal protein S3 (NB4)	Structural component of the 40S ribosomal subunit; has endonuclease activity; involved in DNA damage repair; interacts with MDM2 resulting in p53 stability.	(1/1)	Y (1/1)	X
P62753	*RPS6*	40S ribosomal protein S6	Structural component of the 40S ribosomal subunit; involved in rRNA processing; involved in the selective translation of a certain class of mRNAs.	(1/1)	Y (1/1)	
P62081	*RPS7*	40S ribosomal protein S7	Structural component of the 40S ribosomal subunit; involved in rRNA processing/maturation; binds 3’-UTR and 5’-UTR of mRNA; involved in translation initiation.	(2/2)	Y (2/2)	
P62241	*RPS8*	40S ribosomal protein S8 (NB4)	Structural component of the 40S ribosomal subunit; involved in the maturation of the 18S rRNA.	(1/1)	N	
Q14684	*RRP1B*	Ribosomal RNA processing protein 1 homolog B	Acts as a transcriptional coactivator; involved in mRNA splicing; promotes RNA pol II transcription; involved in rRNA processing.	(3/5)	Y (1/1)	
O76021	*RSL1D1*	Ribosomal L1 domain-containing protein 1	Involved in large subunit rRNA processing/maturation; inhibits PTEN translation.	(4/8) (m)	Y (2/4) (m)	
Q9Y265	*RUVBL1*	RuvB-like 1	ATP-dependent DNA helicase; component of the NuA4 histone acetyltransferase complex; binds to the TF-IID transcription complex; involved in H2A and H4 acetylation and RNA pol II transcriptional activation; involved in C/D snoRNP assembly; has a role in DNA repair; required for MYC oncogenesis.	(3/6) (m)	N (m)	
Q9Y230	*RUVBL2*	RuvB-like 2	ATP-dependent DNA helicase; component of the NuA4 histone acetyltransferase complex; binds to the TF-IID transcription complex; involved in H2A and H4 acetylation and RNA pol II transcriptional activation; involved in C/D snoRNP assembly; has a role in DNA repair; binds β-catenin; required for MYC oncogenesis; suppresses expression of ATF2 and endoplasmic reticulum stress response genes.	(4/4)	Y (2/2)	
Q13435	*SF3B2*	Splicing factor 3B subunit 2	Part of the SF3B complex; involved in pre-mRNA splicing.	(4/4)	Y (1/1)	
Q15393	*SF3B3*	Splicing factor 3B subunit 3 (NB4)	Part of the SF3B complex; involved in pre-mRNA splicing.	(2/2)	N	
P23246	*SFPQ*	Splicing factor, proline- and glutamine-rich (NB4)	DNA/RNA-binding protein; essential for spliceosome complex formation; enhances RNA pol II transcription; involved in alternative splicing.	(4/4)	Y (3/3)	
O60264	*SMARCA5*	SWI/SNF-related matrix-associated actin-dependent regulator of chromatin subfamily A member 5	DNA-binding helicase; represses rDNA transcription.	(4/4)	N	
Q7KZF4	*SND1*	Staphylococcal nuclease domain-containing protein 1 (NB4)	Transcriptional coactivator of STAT5 and STAT6; mediates miRNA decay.	(2/3)	Y (1/1)	
P08579	*SNRPB2*	U2 small nuclear ribonucleoprotein B	Associated with the U2 snRNP involved in pre-mRNA splicing.	(1/1) (m)	Y (1/1) (m)	
Q07955	*SRSF1*	Serine/arginine-rich splicing factor 1	Involved in regulating the accuracy of splicing and alternative splicing by preventing exon skipping; associates with U1 snRNP and U2AF; involved with mRNA nuclear-cytoplasmic export.	(13/14)	Y (13/14)	
Q01130	*SRSF2*	Serine/arginine-rich splicing factor 2	Required for pre-mRNA splicing; facilitates U1 and U2 snRNP association with pre-mRNA; links 5’ and 3’ splice site components U1 snRNP and U2AF, respectively; regulates alternative splicing; facilitates mRNA export from the nucleus; acts as a transcriptional corepressor.	(27/27)	Y (12/12)	
P12270	*TPR*	Nucleoprotein TPR	Component of the nuclear pore; involved in protein and RNA export/import.	(9/9)	Y (2/2)	
P08670	*VIM*	Vimentin	Involved with LARP6 to stabilize certain mRNAs.	(1/3)	Y (1/3)	X
O95218	*ZRANB2*	Zinc finger Ran-binding domain-containing protein 2	Involved in alternative splicing by modifying 5’-splice site selection.	(29/33)	Y (10/10)	

Proteins identified by AP-MS/MS analysis using either Spectrum Mill or Mascot platforms are listed in alphabetical order. Proteins followed by (NB4) are proteins that were also identified in AP-MS/MS analysis of nuclear proteins isolated from ATRA-treated NB4 cells and not untreated NB4 cells. Uniprot/Swissprot database accession number (Acc #); number of sites matching the consensus epitope of the anti-AKT phosphosubstrate antibodies (K/R-x-K/R-x-x-S/T or R-x-x-S/T) over the number of potential AKT sites predicted by Scansite 4.0 at low stringency (AKT pSites; m = Scansite 4.0 predictions conducted at minimal stringency); number of potential AKT phosphorylation sites (Scansite 4.0) known to be phosphorylated (Phosphosite) that match the consensus epitope of the anti-AKT phosphosubstrate antibodies (K/R-x-K/R-x-x-S/T or R-x-x-S/T) over the number of potential AKT phosphorylation sites (Scansite 4.0) known to be phosphorylated (Phosphosite). Proteins that have been demonstrated to be substrates of AKT were marked with an “X” (AKT sub).

**Table 2:** Biogenesis/mRNA processing-related proteins in complex with nuclear PKR.

Acc #	Gene Name	Description	Function	Involvement in Disease
P55265	*ADAR*	Double-stranded RNA-specific adenosine deaminase (1)	Catalyzes the hydrolytic deamination of multiple adenosines to inosines in RNA. This can result in diverse effects as a consequence of RNA modification.	Dyschromatosis hereditaria, Aicardi-Goutieres syndrome 6
Q8WYP5	*AHCTF1*	Protein ELYS	Required for the assembly of a functional nuclear pore complex (NPC). The NPC is required for nuclear-cytoplasmic transport of RNA species and ribonucleoproteins (RNP) complexes and vice versa for the transport of ribosomal proteins (RPs); has effects on RNA pol II activity.	
Q13838	*BAT1*	Spliceosome RNA helicase DDX39B	Component of the THO subcomplex of the TREX complex that specifically associates with spliced mRNA; has a role in the nuclear export of unspliced mRNAs. Weak RNA helicase activity that catalyzes the first step in spliceosome assembly for the subsequent binding of the U2 snRNP.	
Q14692	*BMS1*	Ribosome biogenesis protein BMS1 homolog	Maturation of the 40S ribosomal subunit in the nucleolus; binds U3 snoRNA and may be required for the maturation of rRNA.	Aplasia cutis congenita (ACC)
Q9Y3Y2	*C1orf77*	Chromatin target of PRMT1 protein	Associates with the methylsome complex to induce gene transcription; is a component of the TREX complex; associates upstream of the exon junction complex (EJC) on spliced mRNAs and facilitates their nuclear export.	
Q9BRJ6	*C7orf50*	Uncharacterized protein C7orf50	*Not reported*. Has RNA binding ability; associates with multiple proteins involved in processing of 27S and 18S rRNAs.	
O14646	*CHD1*	Chromodomain-helicase-DNA-binding protein 1	Involved in chromatin remodeling; substrate recognition component of the transcription regulatory histone acetylation (HAT) complex SAGA; regulates RNA polymerase I and II transcription. Associated with diverse mRNA splicing complexes (FACT, PAF and U2 snRNP); blocks DNA replication.	Pilarowski-Bjornsson syndrome (PILBOS)
P38432	*COIL*	Coilin	Major component of Cajal Bodies; involved in the function or assembly/disassembly of nucleoplasmic snRNPs.	
Q92499	*DDX1*	ATP-dependent RNA helicase DDX1	RNA helicase with activity toward RNA-RNA and RNA-DNA helices. Binds poly A mRNA and may be involved in the processing and polyadenylation of the 3’-end of mRNA; involved in tRNA splicing. Acts as a sensor of dsRNA and is involved in the induction of inflammatory cytokines.	
Q8TDD1	*DDX54*	ATP-dependent RNA helicase DDX54	Represses the transcriptional activity of nuclear receptors. Involved in RNA processing.	
Q08211	*DHX9*	ATP-dependent RNA helicase A (1)	RNA-DNA helicase with role in DNA replication, RNA transcription, translation, and RNA silencing; hnRNP actin binding. Transcriptional activator; mediates MYC mRNA stability: Interacts with RELA, IGFBP1, CREB-BP. Involved in viral infection and inflammasome activation; known substrate for EIF2AK2 (PKR).	
Q8IY37	*DHX37*	Probable ATP-dependent RNA helicase DHX37	Has a role in rRNA processing.	
O60832	*DKC1*	H/ACA ribonucleoprotein complex subunit 4	Required for ribosome biogenesis and telomere maintenance; promotes cell to cell and cell to substratum adhesion, increases the cell proliferation rate; catalytic unit of the H/ACA snoRNP complex which is required for pseudouridylation of rRNA; required for correct processing/trafficking of TERC	X-linked Dyskeratosis Congenita (XDKC), Hoyeraal-Hreidarsson syndrome (HHS)
Q99848	*EBNA1BP2*	Probable rRNA-processing protein EBP2 (1)	Required for the processing of the 27S pre-rRNA; interacts with Ebstein-Barr virus (EBV) EBNA1 protein; required for stable EBV episome segregation	
P19525	*EIF2AK2*	Interferon-induced, dsRNA-activated protein kinase	dsRNA-binding kinase activated in response to diverse stresses; phosphorylates eIF2α leading to inhibition of general translation; activation may favor IRES-mediated translation; phosphorylates p53 to stabilize it; alters MYC isoform expression.	Elevated constitutive activity associated with diverse diseases.
P38919	*EIF4A3*	Eukaryotic initiation factor 4A-III	ATP-dependent RNA helicase; component of a splicing-dependent multiprotein exon junction complex (EJC) deposited at splice junction on mRNAs; affects nuclear-cytoplasmic transport of mRNAs; enhances translation of spliced mRNA.	Richieri-Costa-Pereira syndrome (RCPS)
P56537	*EIF6*	Eukaryotic translation initiation factor 6 (1)	Binds to the 60S ribosomal subunit and prevents its premature association with the 40S ribosomal subunit to form the 80S initiation complex in the cytoplasm; affects 60S ribosomal subunit export from the nucleus; enhances the translation of certain transcription factor mRNAs (CEBP, ATF4); affects miRNA silencing of mRNAs; controls the expression mitochondrial respiratory chain genes.	High expression in colon carcinoma
Q15717	*ELAVL1*	ELAV-like protein 1	Ribonucleoprotein complex; involved in 3′ UTR AU-rich element (ARE) dependent MYC, FOS, and IL3 stabilization; binds p53 mRNA to facilitate its export from the nucleus.	
Q9BVP2	*GNL3*	Guanine nucleotide-binding protein-like 3 (1)	Stabilizes MDM2 by preventing its ubiquitination, and proteasomal degradation.	
O60812	*HNRNPCL1*	Heterogeneous nuclear ribonucleoprotein C-like 1 (1)	Ribonucleosome component affecting hnRNPs.	
Q14103	*HNRNPD*	Heterogeneous nuclear ribonucleoprotein D0	Component of the ribonucleosomes; binds 3′ AU-rich elements (AREs) of mRNA to destabilize transcripts, binds ssDNA, and can act as a transcription factor; involved in coupled mRNA translation and turn-over.	
P52272	*HNRNPM*	Heterogeneous nuclear ribonucleoprotein M	Pre-mRNA binding protein; part of the spliceosome C complex; binds poly (G) and poly (U) stretches; may affect signaling events leading to TNFα, IL-1α, IL6, and IL10.	
P07910	*HNRPC*	Heterogeneous nuclear ribonucleoproteins C1/C2 (1)	Mediates 40S hnRNP particles assembly; binds 5’ and 3’ poly (U) tracks of mRNA affecting their stability and translation; may play a role in spliceosome assembly and influence splicing of mRNAs through early association with pre-mRNA.	
O14979	*HNRPDL*	Heterogeneous nuclear ribonucleoprotein D-like	Transcriptional regulator of DNA; promotes transcriptional activation in differentiated myotubes; binds 3′ UTR AU-rich elements (AREs) in mRNAs; RNA processing.	Muscular dystrophy, limb-girdle, autosomal dominant 3 (LGMDD3)
Q12906	*ILF3*	Interleukin enhancer-binding factor 3	Involved in biogenesis of circular RNAs from back splicing by binding regulatory elements flanking introns; binds AU-rich element of target RNAs; participates in diverse transcriptional and post-transcriptional event; is an EIF2AK2 (PKR) substrate; phosphorylation results in ILF3 release of circular RNAs.	
P52292	*KPNA2*	Importin subunit alpha-2	Functions in nuclear protein import as an adapter protein for nuclear receptor KPNB1; Ran-dependent.	
O00629	*KPNA4*	Importin subunit alpha-4	Functions in nuclear protein import as an adapter protein for nuclear receptor KPNB1; Ran-dependent.	
Q13601	*KRR1*	KRR1 small subunit processome component homolog	Involved in nucleolar processing of pre-18S ribosomal RNA and 40S ribosome biogenesis.	
Q6PKG0	*LARP1*	La-related protein 1 (1)	Regulates the translation of specific mRNAs downstream of mTORC1 signaling; when unphosphorylated associates with 5’ UTRs of 5’TOP mRNAs blocking translation by inhibiting eIF4G binding; phoshorylation by mTORC1 results in dissociation from 5’TOP mRNAs favoring their translation; under favorable growth conditions, association with 3’UTR of most mRNAs favors their translation.	
Q9NX58	*LYAR*	Cell growth-regulating nucleolar protein (1)	Acts as a transcriptional regulator; functions in the processing of 47S rRNA to 18S and 28S rRNAs; part of the 90S, 60S, and 40S RNP complexes, but not polysomes; prevents nucleolin self cleavage.	
O95251	*MYST2*	Histone acetyltransferase KAT7	HBO1 (HAT) complex which has H4-specific acetyltransferase activity, reduced activity toward H3; positive regulator of RNA pol II; promotes p53 transcription.	
Q8NEJ9	*NGND*	Neuroguidin	Translational repression of cytoplasmic polyA element containing transcripts; involved in the maturation of 40S subunits rRNA.	
Q8WTT2	*NOC3L*	Nucleolar complex protein 3 homolog (1)	Specifically influences RNA pol II transcriptional activity.	
Q9UGY1	*NOL12*	Nucleolar protein 12	Binds 28S rRNA; involved in rRNA processing; stabilizes the nucleus; inhibits apoptosis.	
P46087	*NOP2*	Putative ribosomal RNA methyltransferase NOP2 (1)	S-adenosyl-L-methionine dependent methyltransferase that catalyzes the methylation of cytosine 4447 in 28S rRNA; affects 60S subunit assembly; regulates RNA pol II-mediated transcription; associated with cell proliferation.	
P06748	*NPM1*	Nucleophosmin (1)	Involved in cellular division, ribosome biogenesis and ribosomal export; regulates p53 and p14^ARF^; enhances MYC transcriptional activity; involved in assembly and export of the 40S and 60S ribosomal subunits; negatively regulates EIF2AK2 (PKR).	Myelodysplastic syndromes (MDS) Leukemia, non-Hodgkin’s lymphoma
Q9UQ80	*PA2G4*	Proliferation-associated protein 2G4	Inhibits transcription of some E2F1-regulated promoters by sequestering the HAT complex; associates with 28S 18S and 5.8S rRNAs and U3 snRNAs; involved in the intermediate and late stages of rRNA maturation; mediates cap-independent translation of specific viral IRES containing mRNAs.	
Q9BY77	*POLDIP3*	Polymerase delta-interacting protein 3	Positive regulation of translation; recruits p70 S6 kinase to the ribosome; involved in mRNA export; associates with spliced RNA-protein complexes favoring translation of spliced mRNAs.	
P62136	*PPP1CA*	Serine/threonine-protein phosphatase PP1-alpha catalytic subunit	Protein phosphatase 1 (PP1) is essential for cell division, and participates in the regulation of glycogen metabolism, muscle contractility, and protein synthesis, cell migration; dephosphorylates a variety of substrates including eIF2α.	
P63244	*RACK1*	Receptor of activated protein kinase C1	Scaffolding protein; binds to and stabilizes activated protein kinase C (PKC), increasing PKC-mediated phosphorylation of EIF6 causing its dissociation from the 60S ribosomal subunit; inhibits Src kinases, prolongs G_1_/G_0_, inhibits Wnt signaling, promotes BAX oligomerization; binds HIV NEF1.	Elevated expression in hepatocellular carcinoma
Q09028	*RBBP4*	Histone-binding protein RBBP4	Component of the chromatin assembly factor 1 (CAF-1) complex, which is required for chromatin assembly following DNA replication and DNA repair; the core histone deacetylase (HDAC) complex, which promotes histone deacetylation and consequent transcriptional repression; the nucleosome remodeling and histone deacetylase complex (the NuRD complex), which promotes transcriptional repression by histone deacetylation and nucleosome remodeling; the PRC2/EED-EZH2 complex, which promotes repression of homeotic genes during development; and the NURF (nucleosome remodeling factor) complex.	
P39023	*RPL3*	60S ribosomal protein L3	Component of the large ribosomal subunit; binds 5S rRNA.	
P27635	*RPL10*	60S ribosomal protein L10	Component of the 60S ribosomal subunit; may have an active role in translation initiation; has a role in the negative regulation of RNA pol II.	Autism, X-linked 5 (AUTSX5)
Q96L21	*RPL10L*	60S ribosomal protein L10-like (1)	Component of the 60S ribosomal subunit; may play a role in compensating for the inactivated X-linked gene during spermatogenesis.	
P35268	*RPL22*	60S ribosomal protein L22	Component of the 60S ribosomal subunit; may have a role in translation initiation; binds Ebstein–Barr virus (EBV) EBER transcripts and heparin.	
P62829	*RPL23*	60S ribosomal protein L23	Component of the 60S ribosomal subunit; associates with rRNA; negatively regulates RNA pol II transcription; negative regulation of ubiquitin protein ligase activity;	
P61254	*RPL26*	60S ribosomal protein L26	Component of the 60S ribosomal subunit; involved in rRNA processing; involved in translation initiation; involved in DNA damage response favoring p53-dependent transcription; associates with the 5’ UTR of mRNAs.	Diamond–Blackfan anemia, type 11
P46776	*RPL27A*	60S ribosomal protein L27a	Component of the 60S ribosomal subunit; involved in translation initiation; binds RNA.	
P62910	*RPL32*	60S ribosomal protein L32	Component of the 60S ribosomal subunit; involved in translation initiation; binds RNA.	
P49207	*RPL34*	60S ribosomal protein L34	Component of the 60S ribosomal subunit; involved in translation initiation; binds RNA; binds cadherin.	
P42766	*RPL35*	60S ribosomal protein L35	Component of the 60S ribosomal subunit; involved in the maturation of the 60 S subunit rRNA; involved in translation initiation; binds mRNA.	
Q9Y3U8	*RPL36*	60S ribosomal protein L36	Component of the 60S ribosomal subunit; involved in translation initiation; binds RNA.	
P46783	*RPS10*	40S ribosomal protein S10	Component of the 40S ribosomal subunit; involved in translation initiation; binds RNA.	Diamond–Blackfan anemia type 9
Q9NQ39	*RPS10L*	Putative 40S ribosomal protein S10-like	Component of the 40S ribosomal subunit; localized to the cytosol only; may result from a pseudogene.	
P39019	*RPS19*	40S ribosomal protein S19	Component of the 40S ribosomal subunit; required for pre-rRNA processing and maturation of 40S ribosomal subunits; involved in translation initiation; binds RNA; protein kinases and fibroblast growth factor (FGF); involved in NOTCH signaling.	Diamond–Blackfan anemia, type 1; highly expressed in colon carcinoma.
P62854	*RPS26*	40S ribosomal protein S26	Component of the 40S ribosomal subunit; involved in translation initiation; binds RNA and mRNA; binds cadherin; negatively regulates pre-mRNA splicing;	Diamond–Blackfan anemia, type 10
P56182	*RRP1*	Ribosomal RNA processing protein 1 homolog A	RNA binding protein critical to the generation of 28S rRNA.	
Q9Y3B9	*RRP15*	RRP15-like protein	Involved in rRNA processing.	
Q96EU6	*RRP36*	Ribosomal RNA processing protein 36 homolog	Involved in the early processing steps of the pre-rRNA in maturation pathway leading to the 18S rRNA; involved in the cleavage to liberate 18S rRNA.	
Q9UHA3	*RSL24D1*	Probable ribosome biogenesis protein RLP24	Involved in the biogenesis of the 60S ribosomal subunit; insures NOG1 docking to 60S ribosomal subunit; structural component of the ribosome; involved in translation.	
Q9Y265	*RUVBL1*	RuvB-like 1	ATP-dependent DNA helicase; component of the NuA4 histone acetyltransferase complex; binds to the TF-IID transcription complex; involved in H2A and H4 acetylation and RNA pol II transcriptional activation; involved in C/D snoRNP assembly; has a role in DNA repair; required for MYC oncogenesis.	
Q9Y230	*RUVBL2*	RuvB-like 2	ATP-dependent DNA helicase; component of the NuA4 histone acetyltransferase complex; binds to the TF-IID transcription complex; involved in H2A and H4 acetylation and RNA pol II transcriptional activation; involved in C/D snoRNP assembly; has a role in DNA repair; binds β-catenin; required for MYC oncogenesis; suppresses expression of ATF2 and endoplasmic reticulum stress response genes.	
P28370	*SMARCA1*	Probable global transcription activator SNF2L1 (1)	Chromatin remodeling DNA-binding protein; positively regulates RNA pol II transcription.	
P51532	*SMARCA4*	Transcription activator BRG1	Chromatin remodeling DNA-binding protein; associates with p53; associates with long non-coding RNAs; acts as a transcriptional coactivator or corepressor; positive regulation of RNA pol II pre-miRNA transcription.	Rhab doid tumors and Coffin-Siris syndrome 4 (CSS4)
O60264	*SMARCA5*	SWI/SNF-related matrix-associated actin-dependent regulator of chromatin subfamily A member 5	Chromatin remodeling DNA-binding protein; represses RNA pol-dependent transcription of rDNA; regulates chromatin silencing by recruiting DNA methyltransferases to stretches of rDNA.	
Q92922	*SMARCC1*	SWI/SNF complex subunit SMARCC1	Chromatin remodeling DNA-binding protein; component of the SWI/SNF chromatin remodeling complex; acts as a positive regulator of RNA pol II-dependent transcription; may repress certain genes.	
Q8TAQ2	*SMARCC2*	SWI/SNF complex subunit SMARCC2	Chromatin remodeling DNA-binding protein; component of the SWI/SNF chromatin remodeling complex; acts as a positive regulator of RNA pol II-dependent transcription; may repress certain genes.	
P09661	*SNRPA1*	U2 small nuclear ribonucleoprotein A’	Part of the spliceosome complex involved in pre-mRNA splicing; part of the U2 snRNP; associates with U2 snRNA.	
P62316	*SNRPD2*	Small nuclear ribonucleoprotein Sm D2	Core component of the SMN-Sm complex that mediates snRNP assembly, which occurs in the cytoplasm; facilitates nuclear import of snRNPs; is a component of the U1, U2, U4, and U5 snRNPs; binds RNA; required for pre-mRNA splicing; auto-antibodies found in lupus.	
P62318	*SNRPD3*	Small nuclear ribonucleoprotein Sm D3	Core component of the SMN-Sm complex that mediates snRNP assembly, which occurs in the cytoplasm; facilitates nuclear import of snRNPs; is a component of the U1, U2, U4, and U5 snRNPs; binds RNA; required for pre-mRNA splicing; binds to the downstream cleavage product (DCP) of histone pre-mRNA in a U7 snRNP dependent manner; auto-antibodies found in lupus.	
P18583	*SON*	Protein SON	mRNA splicing cofactor; enhances splicing of mRNAs with weak splice sites, including many cell cycle and DNA repair genes, through the interaction with SRSF2 and RNA pol II.	ZTTK syndrome
O15042	*SR140*	U2 snRNP-associated SURP motif-containing protein	RNA binding; involved in mRNA splicing.	
Q8IYB3	*SRRM1*	Serine/arginine repetitive matrix protein 1	Part of pre- and post-splicing multiprotein mRNP complexes; links sequence-specific splicing factors with snRNP factors of the splicesome; involved in mRNA nuclear export.	
Q01130	*SRSF2*	Serine/arginine-rich splicing factor 2	Required for pre-mRNA splicing; facilitates U1 and U2 snRNP association with pre-mRNA; links 5’ and 3’ splice site components U1 snRNP and U2AF, respectively; regulates alternative splicing; facilitates mRNA export from the nucleus; acts as a transcriptional corepressor.	
Q13247	*SRSF6*	Serine/arginine-rich splicing factor 6	Plays a role in constitutive splicing and can modulate the selection of alternative splice sites; involved in mRNA export from the nucleus.	
Q08945	*SSRP1*	FACT complex subunit SSRP1	DNA-binding component of the FACT complex; involved in RNA pol II mRNA elongation, DNA replication, and DNA repair; may influence p53 stability; auto-antibodies found in lupus.	
P53999	*SUB1*	Activated RNA polymerase II transcriptional coactivator p15	RNA and DNA binding protein that acts as a coactivator of TAF complex-mediated RNA polymerase II transcription.	
O75683	*SURF6*	Surfeit locus protein 6	RNA and DNA binding protein likely involved in ribosome biogenesis and assembly.	
O60506	*SYNCRIP*	Heterogeneous nuclear ribonucleoprotein Q	An hnRNP implicated mRNA processing; three isoforms function diversely; involved in splicing and mRNA turnover; inhibits translation; promotes MYC mRNA stability; part of APOB editsome; may facilitate cytoplasmic vesicle transport of RNAs.	
Q92804	*TAF15*	TATA-binding protein-associated factor 2N	An RNA and ssDNA binding protein with RNA pol II-mediated transcription promoting characteristics.	Extra skeletal myxoid chondrosarcoma
Q8NI27	*THOC2*	THO complex subunit 2	A component of the THO complex that is a sub-component of the TREX complex; associates with spliced and polyA mRNA to export then from the nucleus; required for the release of mRNA from nuclear speckles.	Mental retardation, X-linked 12 (MRX12)
P62995	*TRA2B*	Transformer-2 protein homolog beta	Sequence-specific RNA-binding protein that participates in the control of pre-mRNA splicing; can promote or inhibit exon inclusion.	
Q01081	*U2AF1*	Splicing factor U2AF 35 kDa subunit	Constitutive and enhancer-dependent splicing by mediating protein–protein interactions and protein–RNA interactions required for accurate 3’-splice site selection; facilitates mRNA nuclear export.	Myelodysplastic syndromes (MDS)
P26368	*U2AF2*	Splicing factor U2AF 65 kDa subunit	Has a role in splicing of and 3’ processing of pre-mRNA; required for mRNA export; may link the processes of transcription termination, polyadenylation and export.	
Q9NQZ2	*UTP3*	Something about silencing protein 10	Role in the structure of silenced chromatin; has a role in 40S subunit rRNA processing.	
Q15061	*WDR43*	WD repeat-containing protein 43	Ribosome biogenesis factor; required for RNA pol I-mediated transcription; involved in pre-18S rRNA processing.	
P67809	*YBX1*	Nuclease-sensitive element-binding protein 1	pre-mRNA alternative splicing regulation; stabilizes cytoplasmic mRNAs; promotes the interaction of mRNA with translation initiation factors; acts a transcription factor influencing the expression of numerous genes through RNA pol II-specific means; possesses endonuclease activity; promotes MYC mRNA stability.	

Acc. # refers to the identifier in the UniProtKB-SwissProt. Gene name is that used by the UniProtKB-SwissProt database. Function and Involvement in disease were retrieved in UniProtKB database. (1) Indicates proteins that have been grouped with another protein in the dataset, due to the fact they share peptides identified (mainly because they are isoforms, homologous proteins).

## Abbreviations

AMP	Adenosine Monophosphate
ATF	Activating Transcription Factor
ATP	Adenosine Triphosphate
BMFD	Bone Marrow Failure Disorder
DBA	Diamond-Blackfan Anemia
DC	Dyskeratosis Congenita
eIF	Eukaryotic Translation Initiation Factor
eEF	Eukaryotic Translation Elongation Factor
FGF	Fibroblast Growth Factor
GDP	Guanosine Diphosphate
GTP	Guanosine Triphosphate
GTPase	Guanosine Triphosphatase
HAT	Histone Acetyltransferase
hnRNP	Heterogeneous Nuclear Ribonucleoprotein
IFN	Interferon
IL	Interleukin
IRES	Internal Ribosome Entry Site
MDS	Myelodysplastic Syndrome
mRNA	Messenger RNA
ncRNA	Non-coding RNA
PIC	Pre-Initiation Complex
PtdIns	Phosphatidylinositol
RNP	Ribonucleoprotein
RP	Ribosomal Protein
RPL	60S Ribosomal Subunit Protein
RPS	40S Ribosomal Subunit Protein
rDNA	Ribosomal DNA
rRNA	Ribosomal RNA
scaRNA	Small Cajal Body RNA
SDS	Shwachman-Diamond Syndrome
snRNA	Small Nuclear RNA
snRNP	Small Nuclear Ribonucleoprotein
snoRNA	Small Nucleolar RNA
TCS	Treacher-Collins Syndrome
TNFα	Tumor Necrosis Factor-Alpha
TRRAP	Transformation/Transcription Domain-Associated Protein
tRNA	Transfer RNA
uORF	Upstream Open Reading Frame
UPC	Ubiquitin Proteosome Complex
UPR	Unfolded Protein Response
UTR	Untranslated Region
VEGF	Vascular Endothelial Growth Factor
